# S-adenosylmethionine as an epigenetic treatment of depression in adults with childhood trauma

**DOI:** 10.1080/17501911.2026.2645001

**Published:** 2026-04-24

**Authors:** Anne Alkema, Winni Schalkwijk, Evelien Bohte, Luc Draisma, Astrid Hoppe, Charlotte Koch, Veerle Refuge, Birgit Romberg, Jurjen J. Luykx, Judith J.M. Jans, Wiepke Cahn, Eline Regeer, Marco P.M. Boks

**Affiliations:** a Department of Psychiatry, Brain Center University Medical Center Utrecht, University Utrecht, Utrecht, The Netherlands; b Department of Emergency Psychiatry, Arkin Mental Health Care, Amsterdam, The Netherlands; c Department of Common Mental Health Disorders, Altrecht GGZ, Utrecht, The Netherlands; d Department of Pharmacy, University Medical Center Utrecht, Utrecht, The Netherlands; e Department of Psychiatry, Amsterdam UMC, Amsterdam, The Netherlands; f Department of Altrecht Science, GGZ inGeest, Amsterdam, The Netherlands; g Department of Psychiatry and Neuropsychology, Maastricht University Medical Center, Maastricht, The Netherlands; h Department of Genetics, Section Metabolic Diagnostics, University Medical Center Utrecht, Utrecht, The Netherlands; i Department of Bipolar Disorders, Altrecht GGZ, Utrecht, The Netherlands; j Altrecht Science, Altrecht GGZ, Utrecht, The Netherlands; k Department of Mood Disorders, Dimence Specialised Mental Health, Deventer, The Netherlands

**Keywords:** Epigenomics, S-adenosylmethionine, depressive disorder, adverse childhood experiences, clinical trial

## Abstract

**Background:**

Childhood trauma is associated with increased risk of depression and epigenetic alterations in stress-related pathways. Preclinical studies suggest that methyl donors may facilitate DNA methylation changes. This first-in-human trial of epigenetic treatment investigated methyl donor S-adenosylmethionine (SAMe) as add-on to trauma-focused therapy for depression.

**Methods:**

In this randomized, double-blind, placebo-controlled trial with 6-month follow-up, 31 adults with trauma-related depression were enrolled. Participants received 1200 mg SAMe or placebo alongside 12-weeks trauma-focused therapy. Depression symptoms were assessed using the Hamilton Rating Scale of Depression (HAM-D). Plasma SAMe levels and genome-wide DNA methylation were measured pre- and post-treatment, including epigenome-wide association analyses, differentially methylated region analyses, and epigenetic clock measures.

**Results:**

Twenty-eight participants completed the study. Depression symptoms decreased significantly during treatment and remained improved at follow-up, reflecting the effect of psychotherapy, without clinical benefit of SAMe. SAMe supplementation did not alter plasma SAMe levels. However, SAMe treatment was associated with differential methylation across 66 regions. Epigenetic clock analyses showed no consistent treatment-related changes.

**Conclusions:**

This first-in-human epigenetic intervention study in psychiatry demonstrates the feasibility of combining trauma-focused psychotherapy with targeted epigenetic analyses. While SAMe showed no additive clinical effects, the results provide important leads for future trials.

**Clinical trial identifier (EudraCT):**

2017-002097-38.

## Plain Language Summary

Depression is common and can be especially difficult to treat in people who experienced traumatic events during childhood. Early life stress may leave long-lasting biological “marks”: small changes to DNA that influence how genes work, in a process called “DNA methylation.” Trauma induces DNA methylation changes around genes related to the stress response, which can affect how the body responds to stress.

In this study, we tested whether a natural substance called S-adenosylmethionine (SAMe) could help improve depression symptoms by influencing these biological traces. We investigated whether using SAMe alongside trauma-focused psychotherapy could help facilitate treatment-induced changes in DNA methylation linked to stress.

Twenty-eight adults with depression and a history of childhood trauma participated in this clinical trial. Half received SAMe and the other half received a placebo. All participants received trauma-focused psychotherapy. We measured depression symptoms and DNA methylation patterns before and after treatment. Depression symptoms were improved after psychotherapy in both groups, but SAMe did not lead to additional improvement in symptoms. We also did not observe clear changes in DNA methylation. However, some small, regional differences were detected, that can inform future research directions.

Although SAMe did not show clear added benefit in this study, the trial provides important insights into the challenging translation of biological treatments from laboratory research into clinical practice. It also offers guidance for designing future studies that aim to target biological processes involved in trauma-related depression.

## 1. Introduction

Major depression is the most prevalent psychiatric disorder, severely impairing psychosocial functioning and quality of life [1,2]. The occurrence of childhood trauma contributes to the etiology of depression [3,4] and increases the likelihood of antidepressant treatment failure [5–7]. Evidence suggests that childhood trauma alters both psychological development and neurobiological systems, leading to distinct pathophysiological mechanisms in depression [8,9]. Trauma-focused psychotherapies have shown promise in reducing depression symptoms, when incorporated into depression treatment for patients with childhood adversity [10–12]. However, it remains unclear whether current treatments sufficiently address the long-lasting role of childhood adversity in the risk and course of depressive disorders [13,14]. The efficacy of pharmacological and psychological antidepressant treatment remains suboptimal in more than one-third of patients [1]. Therefore, further research is warranted to develop more effective treatments for individuals with depressive disorders and a history of childhood trauma.

Increased understanding of the biological basis of the relationship between childhood trauma and depression is providing insights that may inform the development of new treatments. One approach could be based on the emerging evidence that severe stress during development has enduring effects on brain function [15,16] that may be related to epigenetic changes that are involved in the long-term clinical effects of childhood trauma [17–19]. DNA methylation (DNAm) is an epigenetic process by which methyl groups are added to the DNA. This process can modify chromatin structure and gene expression without altering the DNA sequence. Epigenetics plays a central role in transcriptional regulation, and altered epigenetic processes account for a significant proportion of the physiological changes that underlie altered stress responsiveness [20,21] and risk for mood disorders [22]. Preclinical studies show a consistent link between early life environment, DNAm alterations, and adult stress reactivity and behavior [23]. In humans, long-term effects of childhood trauma stably altering DNA methylation patterns are widely supported [20,24,25]. Notably, DNAm changes induced by treatment of psychological trauma are associated with increased resilience and clinical treatment efficacy [26–28].

Epigenetic alterations related to childhood trauma form the basis of preclinical studies on influencing DNA methylation as a treatment. Foundational work established SAMe as the universal methyl donor for DNA methylation, leading to the proposal of epigenetic modulation using methyl donors as a therapeutic strategy [29–31]. SAMe is involved in multiple biochemical pathways, including the biosynthesis of neurotransmitters [32,33]. In peripheral blood and cerebrospinal fluid (CSF), SAMe concentration ranges in normal and disease states have been established [34]. Interestingly, abnormal cerebrospinal fluid (CSF) levels of SAMe have been reported in depression, suggesting that disruptions in SAMe levels may contribute to its pathophysiology.

Preclinical studies further suggest that DNAm changes induced by methyl donors are conditional on chromatin activation. SAMe is irreversibly metabolized in the liver to S-adenosylhomocysteine (SAH), a potent inhibitor of methyltransferases. Studies in animal models have demonstrated that providing potent methyl donors such as SAMe, alter the steady state of methylation [32,34–36]. Accordingly, SAMe deprived diets lead to hypomethylation [34]. SAMe adjusts DNA methylation in human neuroblastoma SK-N-SH and colorectal cancer cell cultures [37,38], inhibits *in vitro* tumorigenesis [39,40], and is capable of altering DNA transcription [41]. In the acute stress-responsive gene expression, low levels of SAMe led to decreased adaptive responses upon exposure to stress in *Caenorhabditis elegans* [42]. Saunderson et al. demonstrated that SAMe altered DNA methylation in stress response genes only when SAMe supplementation was combined with the forced swim stressor [43], supporting the idea that changes in DNA methylation are most likely to occur in open chromatin regions [33,44,45].

Clinical research demonstrates that either oral or parenteral administered SAMe crosses the blood-brain barrier and increases CSF levels, including in patients with neuropsychiatric conditions [32,46]. Electroencephalography (EEG) research found changes in EEG patterns after SAMe administration typical of classical antidepressants [47,48]. Several studies have been conducted on the use of oral SAMe at doses up to 1600 mg per day, demonstrating that SAMe is a safe supplement with no severe side-effects [49]. Clinical trials found positive contributions of SAMe in comparison to placebo in the treatment of depression independent of any epigenetic effects [50–54]. SAMe is used as a prescription drug in several European countries [55], and is registered as a dietary supplement for mood enhancement by the Federal Drug Administration (FDA) in the United States of America. However, to the best of our knowledge, SAMe has never been clinically investigated for its potential antidepressant treatment effects by targeting epigenetic regulation.

Importantly, childhood trauma and chronic stress have been associated with accelerated epigenetic aging, suggesting that early adversity may have lasting effects on biological aging processes [56]. It is well established that DNAm patterns reliably identify biological aging through epigenetic clocks, which estimate biological age based on methylation levels at specific CpG sites [57]. While epigenetic clocks do not represent specific mechanistic targets, they provide integrative measures of cumulative methylation changes across multiple biological pathways. Furthermore, interventions targeting stress-related pathways can impact epigenetic age acceleration [58,59].

It is currently unclear whether SAMe is capable of targeting DNA methylation changes in depressed patients with a history of childhood trauma. The documented efficacy of targeted changes in DNA methylation indicates that this approach may represent a significant advance in minimizing the long-term devastating effects of childhood trauma and in the treatment of depressive disorders. The current study pioneered research of *in vivo* epigenetic therapy outside the field of oncology.

This randomized double blind clinical trial examines the effects of SAMe on DNA methylation levels and depressive symptoms in patients who experienced childhood trauma, in conjunction with trauma-focused therapy. The central mechanistic hypothesis of this study is that the methyl donor SAMe supports DNA methylation changes preferentially at stress- and trauma-responsive genes when these loci are transcriptionally activated during psychotherapy. The objective of our study is to investigate whether depression-related symptom improvement is associated with pathway-specific DNA methylation changes rather than global alterations in methylation. Building on evidence that DNA methylation changes occur preferentially in open chromatin, trauma-focused therapy was used to activate stress-response pathways. Epigenetic clock measures were included as secondary outcomes to explore whether therapy-associated methylation changes are reflected in biological aging markers, potentially mitigating the biological aging effects associated with childhood trauma.

## 2. Methods and materials

### 2.1. Study population

In this double-blind randomized controlled pilot trial with a 6-month follow-up period, 31 adults diagnosed with a current depressive episode and exhibiting high levels of childhood trauma were included. The diagnosis of a current depressive episode was determined by a clinician’s assessment and the Structured Clinical Interview for DSM-IV-TR (SCID) [60]. The assessment of childhood trauma was conducted using the Dutch version of the Childhood Trauma Questionnaire (CTQ) [61,62]. The definition of high levels of trauma was based on the cutoff score of moderate to severe for any of the five CTQ subscales: emotional abuse (subscale score ≥ 13), emotional neglect (subscale score ≥ 15), physical abuse (subscale score ≥ 10), physical neglect (subscale score ≥ 10), and sexual abuse (subscale score ≥ 8). These individuals were about to commence trauma-focused psychotherapy in the University Medical Center of Utrecht in the Netherlands. Two participants withdrew from the study and one participant discontinued participation due to a non-study-related serious adverse event. This resulted in a total sample size of *N* = 28. The inclusion criteria consisted of an age between 18–65 years, current depressive episode, ≥1 moderate to severe CTQ subscale score, the capability of providing written informed consent, and stable psychotropic medication use from one month prior to inclusion and during study participation, meaning: no switches in mood stabilizers, antidepressants and antipsychotics. Individuals who met any of the following criteria were excluded from participation: compulsory admission or treatment, rapid cycling bipolar disorder (as defined by four or more mood episodes in the previous 12 months), a major somatic disorder that interfered with treatment or diagnosis, and pregnancy, breastfeeding or anticipated plans for pregnancy during study participation. The initial study protocol included bipolar depression; however changes in study protocol were made to include any type of depression to increase the inclusion rate during the COVID-19 pandemic. Despite broadening inclusion criteria, the study failed to reach its recruitment target of *n* = 100, due to covid delays and expiration of medication batches. Participants were reimbursed for travel expenses and received monetary compensation for completing the follow-up. Written informed consent was obtained from all participants in accordance with procedures approved by the University Medical Center Utrecht ethical committee under number 17-291, and in accordance with the Declaration of Helsinki.

### 2.2. Study procedures

Demographic information was collected at baseline (T0). The 12-week trial period consisted of randomized daily use of either SAMe or placebo in addition to individual trauma-focused psychotherapy sessions. During the 12-week intervention period, all participants received one psychotherapy session per week as part of standard care. After 12 weeks (T1), the study medication was stopped, while the therapy sessions could continue on a need-basis. At T0 and T1, the Hamilton Depression Rating Scale (HAM-D) [63] was used to assess depressive symptoms and treatment effect. Treatment response was defined as 50% reduction or more on the HAM-D score. The Montgomery–Åsberg Depression Rating Scale (MADRS) [64] was administered at T0, T1, and after 6-months of follow-up (T2). Blood samples were drawn at T0 and T1 from all participants. Blood samples at T1 were obtained on the final day of study medication intake. Participants were instructed to take their last morning dose minimally 4 hours prior to the study visit. Blood draws were not time-locked to a fixed number of hours post-ingestion but were conducted during scheduled clinic visits, reflecting pragmatic clinical conditions. From these samples collected in EDTA, we obtained plasma levels of S-adenosylmethionine (SAM) and S-adenosylhomocysteine (SAH), genotypes and DNA methylation (DNAm) levels. At weekly study visits before or after the trauma-focused psychotherapy appointments, a short interview on possible side-effects and pill count took place. Due to color changes of study medication, a recall and investigation were initiated. The investigation concluded that the changes were merely cosmetic. The recall affected three participants in their trail period who after study deblinding appeared to be enrolled in the SAMe group. One participant discontinued SAMe in week 6, the two others in week 9 of the trial period.

### 2.3. Same versus placebo as add-on to trauma-focused psychotherapy

The SAMe and placebo tablets were purchased from biotech company Gnosis by Lesaffre. The daily dose of SAMe consisted of 3 white, oblong tablets of 400 mg each. The target dose of 1200 mg/day was selected based on prior clinical trials demonstrating antidepressant efficacy and favorable tolerability within the 800–1600 mg/day range [46,49]. Participants were instructed to take the tablets once a day, in the morning, and not on an empty stomach. The placebo tablets were precisely matched in appearance and weight. The tablets were film-coated, therefore taste and smell differences due to the active substance were minimized. The SAMe dosage was titrated over a period of 5 days, starting with 400 mg/day for 2 days, then 800 mg/day for 2 days, followed by 1200 mg/day thereafter, for a total of 12 weeks starting simultaneously with trauma-focused treatment. For stopping the reverse schedule was used. The pharmacist BR assigned the use of either SAMe or placebo and blinded and dispensed the study medication to ensure blinding of both participants and research staff. Participants eligible for study participation were seen by a psychiatrist prior to inclusion to ensure a diagnosis of depression, using the SCID as part of standard care, and to align the clinical treatment with study participation. The trauma-focused psychotherapies were individually conducted by certified therapists. Modalities included Eye Movement Desensitization Reprocessing (EMDR) [65], Narrative Exposure Therapy (NET) [66], Imaginal Exposure (IE) [67] and Brief Eclectic Psychotherapy for Posttraumatic Stress Disorder (BEPP) [68] according to standardized Dutch protocols.

### 2.4. Blood plasma analyses

SAM and SAH concentrations in plasma pre- and post-treatment were determined. For detailed methods, see the Supplementary File: SAM/SAH plasma analyses. Due to the recall of study medication, three participants did not have a representative post-treatment plasma sample and were removed from further analyses. In the statistical analyses, one outlier (SD > 3) was omitted.

### 2.5. Epigenetic analyses

#### 2.5.1. Genotyping and DNA methylation quantification

DNA was extracted from whole blood using standard protocol. Genotyping was conducted using the Global Screening Arrays (GSA) Infinum iSelect 24 × 1 HTS Custum BeadChip Kit (Illumina Inc., San Diego, CA). Genome-wide DNA methylation was quantified using the Illumina Infinium Methylation EPIC v2 BeadChip Kit. We preprocessed the dataset in R version 4.3.2 with the *meffil* package [69]. All samples matched their genetic identity using SNP profile included on the array for quality control purposes. One duplicate sample was omitted. The detection p value was set at >0.01 and 1271 bad CpG probes were removed. After quality control, 929,332 probes remained for further analysis. The methylation levels are represented as ’beta’ values, ranging from 0 (indicating no cytosine methylation) to 1 (indicating full cytosine methylation). However, for improved statistical validity, we used the *M*-values, which are the log2 transformations of the beta values [70].

#### 2.5.2. Epigenome wide association studies

To identify differentially methylated probes and regions after intervention, we performed longitudinal analyses. DNAm levels measured post-treatment were used as the outcome in a linear regression model, with randomization to the SAMe or placebo group as the independent variable and DNAm levels pre-treatment and gender were the control variables as previously described [26,71]. The model was thus defined as *methylation post-treatment ~ methylation pre-treatment + randomization group + gender + cell count difference*. Inclusion of pre-treatment methylation levels for each CpG site serves to control for stable interindividual influences on DNA methylation, including age-related and smoking-related differences, given that these factors are unlikely to change meaningfully over the 12-week intervention period. This longitudinal approach reduces residual variance and increases sensitivity to within-individual methylation change. The QQ-plot comparing expected versus observed *p*-values yields a lambda of 1.05, indicating minimal genomic inflation and adequate control of type I error. To obtain maximal statistical power, surrogate variables and genetic principal components were not added to the model. The pre- and post-treatment samples were located on the same arrays, which minimizes batch effects and other potential technical confounding. Genetic ancestry was accounted for since the pre-treatment methylation levels for each participant were used as control variable. Cell-type proportions were estimated from DNA methylation data using the Houseman method and reference panel “*blood gse35069 complete*.” The EWAS models included within-individual changes in estimated cell-type composition (post–pre) for B cells, CD4+ T cells, CD8+ T cells, NK cells, monocytes, neutrophils, and eosinophils, in order to account for treatment-related shifts in leukocyte composition over time. This approach adjusts for cellular composition dynamics, while baseline differences in cell-type composition are implicitly accounted for by inclusion of baseline methylation levels. The *p*-values were adjusted for multiple testing using the false discovery rate (FDR) from the Benjamini – Hochberg method, and FDR <0.05 was considered epigenome-wide significant. Linear regression assumptions were assessed by examining the residual distribution for the identified loci. As sensitivity analysis, we performed an EWAS with a selection of the top quantile probes that captured the most variance to reduce the dimensionality of the data and focus on the most informative probes. Since the SAMe and placebo groups differed in comorbidity occurrence, suggesting they are clinically different groups (see Table S1), we added the number of diagnoses per participant as covariate to the sensitivity model, leading to: *subset methylation post-treatment ~ subset methylation pre-treatment + randomization group + gender + delta cell counts + number of diagnoses.*

Differentially methylated regions (DMRs) were identified based on the *p*-values of individual methylation loci using the *dmrff* package [72]. A DMR was selected when a genomic region had a Bonferroni *p*-value <0.05 and contained >1 CpG site. We analyzed identified differentially methylated regions (DMRs) in 54 samples of 27 individuals. Gene enrichment analyses were performed with the *IlluminaHumanMethylationEPICanno.ilm10b4.hg19* package for annotation and the GOrilla analysis tool that uses the gene ontology (GO) classification system for GO term analysis [73,74]. The GO term analysis was performed for the EWAS results of which CpGs were ranked from the lowest to highest FDR corrected *p*-value. Secondly, the annotated CpGs underlying the DMRs were analyzed as target genes against the annotated genes from the EPIC array as background. The enrichment *p*-value was set at *p* < 10–5 for the single ranked gene lists, and at *p* < 10–3 for the target versus background analyses.

#### 2.5.3. Epigenetic clocks

From the quality controlled DNAm data, epigenetic age was calculated pre- and post-treatment. We used the principal component (PC) versions of four common epigenetic clocks: the Horvath2 skin and blood clock [75], the Hannum clock [76], GrimAge [77], and PhenoAge [75]. PC clocks are versions retrained from DNAm PCs instead of CpG level DNAm data, with improved reliability and detection of intervention effects [78]. In addition, we calculated the pace of aging clock DunedinPACE [79], using the *DunedinPACE* package in R. Epigenetic age acceleration was calculated as epigenetic age residuals, reflecting deviation of epigenetic age from chronological age. Since the DunedinPACE clock already reflects pace of aging regardless of chronological age, we did not use a residualised version of this clock. Bivariate correlations between all clocks and chronological age were calculated. For each of the clocks, we studied whether SAMe influences epigenetic age changes over the treatment period using the model *epigenetic age post-treatment ~ epigenetic age pre-treatment + randomization group + gender*. Secondly, we studied the association between epigenetic age acceleration at baseline with baseline depression symptoms with the model *depression symptoms pre-treatment ~ epigenetic age acceleration pre-treatment + age + gender*. Third, we studied whether between baseline epigenetic age acceleration and changes in epigenetic age from pre- to post-treatment were associated with changes in depression symptoms with the model *changed depression symptoms ~ epigenetic age acceleration pre-treatment + gender*. The assumptions of linear regression were evaluated by examination of the plots of model diagnostics.

### 2.6. Statistical analyses

Differences between patient groups were tested for statistical significance using Fisher’s exact test for categorical variables and the Mann-Whitney U test for continuous variables. For pre- to post-treatment differences in the total group, we used the Wilcoxon signed-rank test. Obtained power and the required effect size was analyzed based on the Mann-Whitney U test using the G*Power software [80]. The significance level for all tests was *p* < 0.05. All analyses were performed using R (version 4.3.2) packages for statistical computing.

## 3. Results

### 3.1. Baseline characteristics

Table S1 presents the demographic characteristics of the participants at the baseline measurement. As illustrated in Table S1, the participants in the placebo group exhibited a significantly higher mean age. Individuals who participated in the SAMe group demonstrated significantly more DSM-IV classifications, as well as a more diverse range of classifications. Four participants did not use psychotropic medications, of whom three were in the placebo group and one was in SAMe group. Participants in the SAMe group were on significantly more psychotropic prescription drugs. All childhood trauma subtypes were present and equally distributed among groups. Of all participants, 53.5% experienced ≥ 3 subtypes of childhood trauma. No statistically significant difference was observed between the SAMe and placebo groups in the number of subtypes of childhood trauma (Mann-Whitney U: W = 108, *p* = 0.63, *r* = 0. 09). The majority of participants engaged in EMDR, and IE and BEPP were not selected.

### 3.2. Changes in depression symptoms

**Figure 1.**
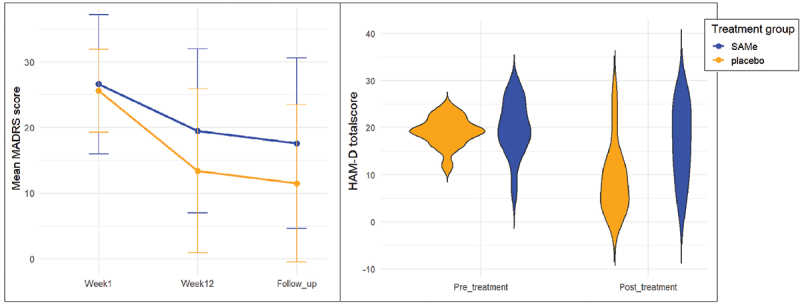
Changes in mean MADRS scores over time (left) and HAM-D total scores pre- and post-treatment (right). Week 1 refers to pre-treatment (T0), week 12 refers to post-treatment (T1), follow-up refers to 6 months post-treatment.

Abbreviations: MADRS = Montgomery-Åsberg Depression Rating Scale; HAM-D = Hamilton Depression Rating Scale; SAMe = S-adenosylmethionine.

The severity of depression symptoms was equally distributed among treatment groups at baseline: the SAMe group showed a mean HAM-D score of 19.8 (SD = 5.79) and the placebo group of 19.1 (SD = 3.07). Furthermore, no significant difference was observed in total MADRS scores at baseline (W = 108, *p* = 0.64). Figure 1 illustrates that within the total sample, depression symptoms significantly decreased from pre- to post-treatment (Wilcoxon signed-rank: V = 314, *p* < 0.01, *r* = 0.66) and persisted through follow-up. Preliminary examination of Figure 1 suggests that the placebo group may outperform the SAMe group. This is evidenced by the delta HAM-D scores, which differ significantly between groups (Mann-Whitney U: W = 45, *p* = 0.01, *r* = 0.45). However, no statistically significant differences in MADRS scores were observed between the SAMe and placebo groups (Mann-Whitney U: W = 61, *p* = 0.16, *r* = 0.27). Calculation of Cohen’s *d* with mean delta HAM-D scores and their standard deviation for the SAMe and placebo groups [81], demonstrated an effect size of −0.96 of SAMe.

### 3.3. Blood plasma analyses and SAMe safety

No significant change was observed in the plasma levels of SAM and SAH for the total group from pre- to post-treatment (Wilcoxon signed-rank: SAM: V = 143, *p* = 0.85, *r* = 0.06; SAH: V = 140, *p* = 0.78, *r* = 0.13). The SAM/SAH ratio also showed no pre-post changes for the total group (Wilcoxon signed-rank: V = 140, *p* = 0.78, *r* = 0.07). The administration of 12 weeks of 1600 mg of SAMe, in comparison to placebo, demonstrated no statistically significant changes in SAM-SAH plasma levels (SAM: Mann-Whitney U: W = 42, *p* = 0.13, *r* = 0.28; SAH: Mann-Whitney U: W = 67, *p* = 1, *r* = 0.09; Supplementary File, Figure S1). The weekly study visits reported high levels of compliance, as evidenced by pill counts, and no drop-outs. The reported adverse effects were similar in frequency across groups (Mann-Whitney U: W = 85, *p* = 0.10, *r* = 0.35) and were generally mild cases of fatigue, headache, and gastro-intestinal complaints.

### 3.4. Epigenome-wide association studies

**Figure 2.**
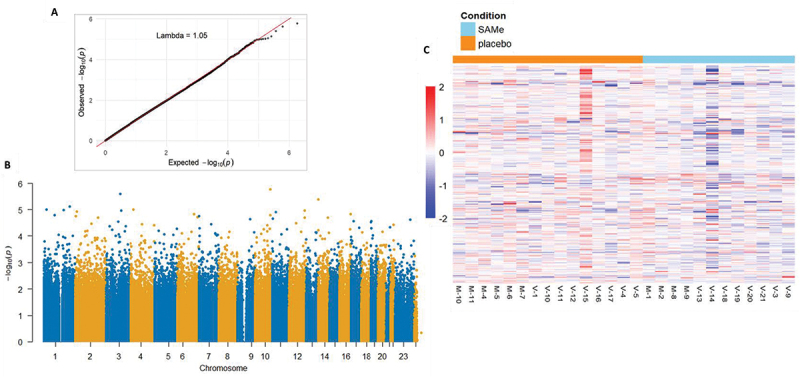
(A) QQ-plot of *p*-values from epigenome-wide association study (EWAS) testing epigenetic treatment with S-Adenosylmethionine (SAMe) versus placebo, in addition to trauma-focused therapy. (B) Manhattan plot depicting probes from the SAMe EWAS. C: Heatplot showing methylation levels in differentially methylated regions (DMRs), in both SAMe (right, light-blue bar) and placebo (left, orange bar) conditions.

Figure 2 illustrates the results of the epigenome-wide association study (EWAS). The use of SAMe was associated with 46,298 nominal differentially methylated probes (DMPs, *p* < 0.05). However, no epigenome-wide significant DMPs were identified following application of a false discovery rate correction (FDR < 0.05). For further details, please refer to the Supplementary File, Table S1, which lists the top 100 probes. A sensitivity analysis using the top quantile probes with the highest variance yielded consistent results: 10,607 nominal DMPs (*p* < 0.05), none of which reached epigenome-wide significance (FDR < 0.05; see Supplementary File, Table S2). This variance-focused sensitivity analysis did not materially alter the overall findings. The full EWAS repository is accessible digitally via the link provided.

The results of the DMR analyses indicated the presence of 66 genomic regions comprising > 1 CpG site with a Bonferroni-adjusted *p*-value of <0.05 (see Supplementary File, Table S3), which may be indicative of regional epigenetic associations with SAMe treatment. An enrichment analysis of the annotated DMPs and DMRs revealed significant but nonspecific pathways (see Supplementary File, Tables S4-S5). The largest DMR, which encompasses the greatest number of differentially methylated CpGs, includes a region of hypomethylation in intron 1 of the GMNT gene (*p* = 1.82E-05). This suggests an increase in regional regulatory activity of SAMe metabolism. As there are no significant epigenome-wide DMPs, the DMR and GO term enrichment analyses remain explorative.

### 3.5. Epigenetic clocks

**Figure 3.**
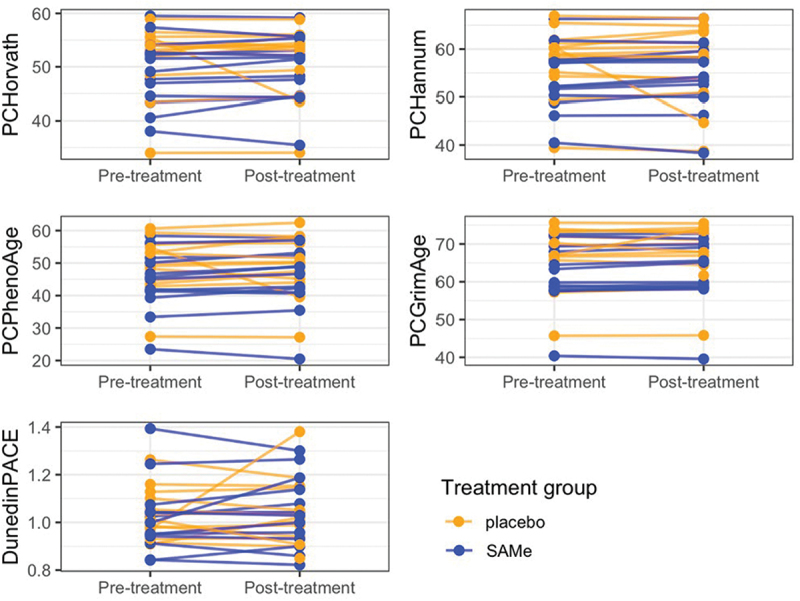
Epigenetic age pre-and post-treatment in the S-adenosylmethionine (SAMe) and placebo group. Principal component (PC) versions of four epigenetic clocks (PCHorvath, PCHannum, PCPhenoAge, PCGrimAge), and the pace of aging clock DunedinPACE.

All four PC epigenetic age measures demonstrated a robust correlation with age in the study population (R ranging from 0.82 to 0.96; see Supplementary File Figure S2). Figure 3 shows no statistically significant differences in epigenetic age in the total group, from pre- to post-treatment (Wilcoxon signed-rank: Horvath V = 148, *p* = 0.34, *r* = –0.22; Hannum V = 153, *p* = 0.40, *r* = –0.19; PhenoAge V = 115, *p* = 0.08, *r* = –0.39; GrimAge V = 122, *p* = 0.11, *r* = –0.35; DunedinPACE V = 171, *p* = 0.68, *r* = –0.10). Linear regression analysis demonstrated that pre-treatment epigenetic age was a significant predictor of post-treatment epigenetic age, as was expected (*p* < 0.01 for all clocks), without a significant effect of SAMe on post-treatment epigenetic age (Horvath *β* = 0.66, *p* = 0.56; Hannum *β* = 1.40, *p* = 0.32; PhenoAge *β* = 1.22, *p* = 0.44; GrimAge *β* = −0.02, *p* = 0.97; DunedinPACE *β* = −0.00, *p* = 0.91; see Supplementary Table S6).

Accelerated epigenetic age at baseline was not significantly related to depression symptoms at baseline (Supplementary File Table S6) and no significant differences were observed between the SAMe and the placebo group (Mann-Whitney U: Horvath W = 119, *p* = 0.19, *r* = 0.31; Hannum W = 109, *p* = 0.40, *r* = 0.20; PhenoAge W = 93, *p* = 0.94, *r* = 0.02; GrimAge W = 92, *p* = 0.98, *r* = 0.01; DunedinPACE W = 82, *p* = 0.68, *r* = –0.10). Changes in epigenetic age from pre- to post-treatment could not predict changes in depression symptoms (see Supplementary File Table S7). A higher epigenetic age acceleration pre-treatment was associated with improvement in depression symptoms over the course of the treatment period for the PCHorvath clock (Horvath *β* = 1.74, *p* = 0.02; Supplementary File Table S8).

## 4. Discussion

This study is the first randomized controlled trial investigating an epigenetic intervention in mental health, specifically targeting DNA methylation in individuals with depression and a history of childhood trauma. Conceptually, childhood trauma is proposed to induce stable DNA methylation changes in stress-responsive genes, contributing to altered stress circuitry and increased vulnerability to depression. In this framework, trauma-focused psychotherapy is expected to activate these stress-related pathways, creating an open chromatin context in which SAMe supplementation may facilitate pathway-specific methylation changes and alleviate depressive symptoms. The study did not identify an additive impact of SAMe on clinical outcomes or global DNA methylation, yet it offers important insights into the feasibility and constraints of epigenetic interventions in humans. Importantly, the absence of additive clinical or global epigenetic effects should not be interpreted as a failure, but as informative in the context of a first-in-human mechanistic trial. The findings allow us to distinguish between pharmacokinetic, pharmacodynamic, and clinical components of SAMe augmentation, thereby clarifying under which biological conditions epigenetic interventions may or may not operate in psychiatric populations.

### 4.1. Pharmacokinetic considerations

The absence of significantly elevated SAMe plasma levels suggests that pharmacokinetic factors may have limited systemic exposure despite adequate dosing and compliance. This highlights translational challenges when moving from preclinical models to human biology. Given the clinical evidence concerning SAMe dosage and the relatively high doses in this study, inadequate dosing seems a less probable explanation [49], just as that low adherence rates are implausible considering the documented high compliance. The participants had morning dosing instructions for the study medication and the post-treatment blood samples were drawn at the same day of the last SAMe intake. The discovered low plasma levels were not expected [82]. However, the evidence for the half-life of orally administered SAMe differs and is limited [83]. Since the SAMe doses were tapered over the last three days of the intervention period, the final dose contained 400 mg of SAMe on the last day, which could result in a lower measurable concentration. Additionally, the bioavailability of SAMe could be lower than expected when tablets prematurely degrade in the stomach, or in case of a high first-pass-effect in the liver. Rapid degradation of SAMe by enzymes after ingestion would have resulted in high SAH levels, which were also not found. Rapid intracellular uptake with limited detectability in plasma remains plausible, since SAMe functions as an intracellular methyl donor. Individual variation in absorption and metabolism (due to genetic or intestinal differences), tissue-specific distribution, measurement variability and drug-interaction effects may also have contributed the SAMe plasma levels detected in this study.

Another consideration is the stability and bioavailability of the SAMe formulation used in this study. The tablets were enteric-coated with methacrylate polymers to provide gastroprotection and allow for immediate release in the small intestine, thereby optimizing absorption. Quality control procedures for pharmaceuticals, as opposed to nutraceuticals, were meticulously followed. Future studies are advised to perform tablet validation to ensure delivery of active SAMe and to address potential variability in bioavailability.

### 4.2. Pharmacodynamic and epigenetic findings

At the level of pharmacodynamics, SAMe did not induce global methylation changes in peripheral blood, nor were epigenome-wide significant individual CpG sites detected after correction for multiple testing. However, region-based analyses identified 66 differentially methylated regions (DMRs), suggesting localized but subtle epigenetic modulation that was not detectable at the single-probe level. The absence of epigenome-wide significant changes contrasts with preclinical evidence of SAMe-induced epigenetic modulation [55]. Given the results of previous studies indicating that psychopharmaceuticals can induce loci-specific DNA methylation changes [84,85], we expected to find pathway-specific DNA methylation changes within stress-related loci. This discrepancy may be explained by the low SAMe plasma levels but could reflect several other factors. First, the heterogeneity of the human methylome; inter-individual genetic variation, baseline variability in trauma-related epigenetic marks, trauma history, and other environmental exposures, may have obscured subtle changes. Second, methylation changes related to childhood trauma might require more time to manifest [86,87]. The epigenetic changes associated with childhood trauma are typically established early in development and are stable enough to last into adulthood [88,89]. This could mean that these methylation patterns are enduring and may not be easily altered by short-term interventions. Also, SAMe induced epigenetic changes may occur delayed after initial SAMe supplementation and require more time to stabilize [87]. The epigenetic clock analyses indicated no significant changes over the 12-week intervention period, which highlights the need for longitudinal investigations into whether such markers can capture subtle treatment-induced effects and the time needed to catch these changes. Finally, peripheral blood DNA methylation may not reflect tissue-specific epigenetic dynamics within brain regions implicated in stress regulation and depression, limiting inference about central pharmacodynamic effects. Nevertheless, DMR analyses showed subtle regional methylation changes associated with the SAMe metabolism, despite the absence of epigenome-wide statistically significant probes.

The absence of global effects but presence of regional changes suggests that SAMe may exert localized and biologically constrained effects rather than broad methylome shifts. These findings warrant replication in larger cohorts to clarify the biological relevance of these regional effects. The most prominent DMR was located within the glycine N-methyltransferase (GNMT) gene, a central regulator of SAMe homeostasis. GNMT catalyzes the transfer of a methyl group from SAMe to glycine, thereby functioning as a key metabolic buffer of intracellular SAMe concentrations, particularly in the liver. Increased GNMT activity can lower circulating SAMe through enhanced methyl group utilization, whereas reduced GNMT function has been associated with elevated SAMe levels [90,91]. In this context, methylation changes at the GNMT locus may reflect adaptive metabolic feedback mechanisms in response to SAMe supplementation and could plausibly relate to the unexpectedly low plasma SAMe concentrations observed in our cohort. However, peripheral blood methylation at GNMT does not directly inform hepatic expression or enzymatic activity, and functional consequences cannot be inferred from the present data. Accordingly, this finding should be regarded as exploratory. Nevertheless, variability in GNMT-mediated SAMe regulation may represent a biologically coherent mechanism contributing to interindividual differences in metabolic response to methyl donor supplementation, warranting targeted investigation in future mechanistic and pharmacokinetic studies. Underlying pathology resulting in liver function variability arising from factors such as obesity, medication use, alcohol consumption, or viral hepatitis [92], may further modulate this pathway. Together, these observations highlight the importance of integrating metabolic phenotyping into future trials of epigenetic augmentation strategies.

### 4.3. Clinical outcomes

Clinically, depression symptoms improved substantially in both treatment arms, indicating that trauma-focused therapy was the primary driver of symptom change in this study. These positive effects persisted through six months of follow-up. No additive antidepressant effect of SAMe was observed under the dosing and treatment conditions used in this study. Trauma-focused therapies (TFT), and particularly EMDR, have demonstrated effectiveness in reducing depression symptoms in individuals with history of childhood trauma with or without PTSD diagnosis [11]. This is consistent with our results. However, the diversity of comorbidities of MDD beyond PTSD, including personality disorders, distinguishes our study from previous and current studies [93]. This calls for further investigation of the potential benefits of trauma-focused psychotherapy in a naturalistic clinical population with multiple comorbidities and a history of severe childhood trauma.

The focus on an epigenetic intervention is one of this study’s primary strengths. Adding to that are the rigorous randomized controlled trial (RCT) design, and the integration of trauma-focused psychotherapy, reflecting a sensitive approach to treating a highly vulnerable population while creating a stressful context receptive to DNA methylation changes [43,44]. However, it is important to acknowledge several limitations, primarily related to the relatively small sample size, which may have constrained the statistical power of the study. The observed between-group effect size for change in HAM-D scores (d = −0.96) corresponded to an achieved power of the current study of 0.66. The sample size was insufficient to allow for the overcoming of differences in DSM-IV classifications and the use of psychotropic medication between SAMe and placebo groups. Although diet can influence DNA methylation, dietary parameters were not considered in this study. The effects of diet on DNA methylation vary across nutrients, tissues, and genomic regions [94]. Due to the within-person approach, whereby systematic bias in changes in diet over 12 weeks between the groups are highly unlikely, and for practical limitations, these effects were not specifically included in the study design. Furthermore, the absence of significant SAMe plasma level changes precludes firm conclusions about its bioavailability and mechanism of action. Also residual confounding cannot be ruled out. Of importance is the fact that the study was designed to include 100 patients based on an estimated effect size of d = 0.8. Due to a combination of circumstances, including the challenges posed by the ongoing COVID-19 pandemic, batch expiration and subsequent logistical difficulties in the international distribution of SAMe tablets, it was necessary to terminate the study prior to achieving the stipulated number of participants.

The safety profile and theoretical basis of SAMe suggest that further investigation into its potential as an adjunct in well-defined subgroups is warranted. Future research should prioritize the implementation of larger, adequately powered trials to validate these preliminary findings. Given the heterogeneity of human responses, we suggest to consider individual variations in trauma histories, such as differences in the type, severity, and chronicity of trauma, as well as the context in which it occurred. These factors may contribute to distinct baseline methylation patterns and thereby influence responsiveness to SAMe supplementation [18,19]. Furthermore, variability in depression symptomatology and coping styles, may interact with both methylation dynamics and clinical outcomes [95,96]. Lifestyle factors, such as diet, exercise, and substance use, should also be considered, as they may influence SAMe metabolism and epigenetic responses [97]. We recommend to optimize the timing of SAMe doses and plasma level measurements, and to include pharmacodynamic assessments and liver function measurements in future studies. Additionally, frequent longitudinal methylation measurements are warranted in order to investigate the time needed to establish methylation changes, their durability and timing and the relationship with an increased resilience on the long term.

## 5. Conclusion

This first-in-human epigenetic intervention trial in psychiatry demonstrates that translating methyl donor strategies from preclinical models to clinical populations is biologically complex. The absence of global methylation changes or additive clinical effects, combined with subtle regional epigenetic signals, highlights the importance of pharmacokinetic dynamics, tissue specificity, and timing in future designs. These findings refine the conceptual framework for epigenetic augmentation strategies and provide practical guidance for subsequent mechanistically informed trials in trauma-associated depression. Continued efforts to refine and integrate epigenetic approaches hold promise for advancing the treatment of depression in individuals with childhood trauma.

## Supplementary Material

SAMe_Supplementary_File.docx

## Data Availability

The data and code used for this study are deposited here: https://doi.org/10.34894/ZKLNDX and are available upon reasonable request.

## Article highlights

- Childhood trauma is associated with epigenetic alterations in stress-related genomic regions and an increased risk of depression.
- S-adenosylmethionine (SAMe) is a potent natural methyl donor with potential to facilitate epigenetic changes upon transcriptional activation.
- This randomized controlled trial tested SAMe as a mechanistically informed epigenetic intervention as an add-on treatment to 12-week trauma-focused psychotherapy in depressed patients with a history of childhood trauma.
- Trauma-focused psychotherapy was associated with significant improvement in depressive symptoms across the total sample, while SAMe augmentation did not provide additive clinical benefit or differential methylation in stress-related genomic regions.
- Region-based analyses identified subtle differentially methylated regions, including within GNMT, suggesting localized metabolic feedback effects.
- This first-in-human epigenetic intervention study underscores the importance of pharmacokinetics, tissue specificity, and timing when translating epigenetic strategies from preclinical models to clinical populations.
