# Supplementary material for: S-adenosylmethionine as an epigenetic treatment of depression in adults with childhood trauma
Source: Epigenomics. 2026 Apr 24;18(4):437–49. doi: 10.1080/17501911.2026.2645001 (PMC13166236; doi:10.1080/17501911.2026.2645001)
Supplement: SAMe_Supplementary_File.docx [file IEPI_A_2645001_SM2525.docx]

**Supplementary File**

- Methods SAM/SAH plasma analyses
- Figure S1: Change in SAM/SAH plasma levels pre- to post-treatment
- Table S1: Epigenome wide association study (EWAS) results, top 100 probes
- Table S2: Sensitivity EWAS results, top 100 probes
- Table S3: Differentially Methylated Regions (DMRs)
- Table S4: GO term enrichment analysis EWAS
- Table S5: GO term enrichment analysis sensitivity DMRs
- Figure S2: Correlation heatmap epigenetic clocks
- Table S6-S8: Linear regression analyses epigenetic clocks

Methods SAM/SAH plasma analyses

Analysis of S-Adenosylmethionine (SAM) and S-Adenosylhomocysteine (SAH) in plasma. Stock solutions of SAM (15 mg/10ml, 2.95 mM, MW: 507.82), SAH (6 mg/10 ml, 1.56 mM, MW: 384.41) and their identical isotopes were prepared in 0.1% formic acid in Milli-Q water and stored at -80 ⁰C. Stock IS solutions: d3-SAM (1 mg/1ml, 1.67mM, MW; 597.61) and d4-SAH (5mg/10ml, 1.29 mM, MW: 388.4). The internal standard solution mixture contained ~ 1.5 µM d3-SAM and d4-SAH.

20 µl plasma (1:25 diluted with Milli-Q water) was pipetted into a 1.5 ml Eppendorf tube. 20 µl of 0.5M Dithiothreitol (DTT) and 20µl IS was added to the same tubes, vortexed and incubated for 10 minutes at room temperature. 100 µl extraction solution (0.1% Formic acid in Methanol) was added to the tubes and vortexed. The samples were centrifuged for 5 minutes at 14000 rpm. The supernatant was then analyzed using a Thermo Scientific Vanquish Flex Duo system coupled to an Orbitrap Exploris 240 high-resolution mass spectrometer (Thermo Fisher Scientific, Bremen, Germany) in positive HESI (heated electrospray ionization) mode. The injection volume was 5 µl and the autosampler was set to 10°C. Chromatographic separation was carried out on a Sunfire C8 column (3.5 µm 4.6x100 mm)( ChromaNik Technologies Inc., Osaka, Japan) at a temperature of 40 °C. Solvent A was 0.1% Formic acid in Methanol/Milli-Q water, (5/95 v/v) and solvent B was 0.1% Formic acid in Methanol. The following gradient, with a flow rate of 0.75 ml/min, was used: isocratic 0% B from 0 to 0.3 min, linear from 0 to 100% B during 0.3 to 2.0 min, isocratic 100% B from 2.0 to 4.0 min, linear from 100 to 0% B during 4.0 to 4.2 min, isocratic 0% B (initial solvent conditions) from 4.2 to 7.0 min to equilibrate the column. Analytes were recorded via a full scan with a mass resolving power of 120,000 over a mass range from 375-410 m/z (Normalized AGC target: 20%, RF lens: 75%, max injection time: Auto). Ion source parameters were set to the following values: spray voltage: 3500 V (positive mode), sheath gas: 50 psi, auxiliary gas: 15 psi, sweep gas: 0 psi, ion transfer tube temperature: 350°C, vaporizer temperature: 400°C. Data were acquired using Xcalibur software (version 3.0; Thermo Fisher Scientific). For the integration of raw data peaks, TraceFinder 4.1 software (Thermo Fisher Scientific) was used.


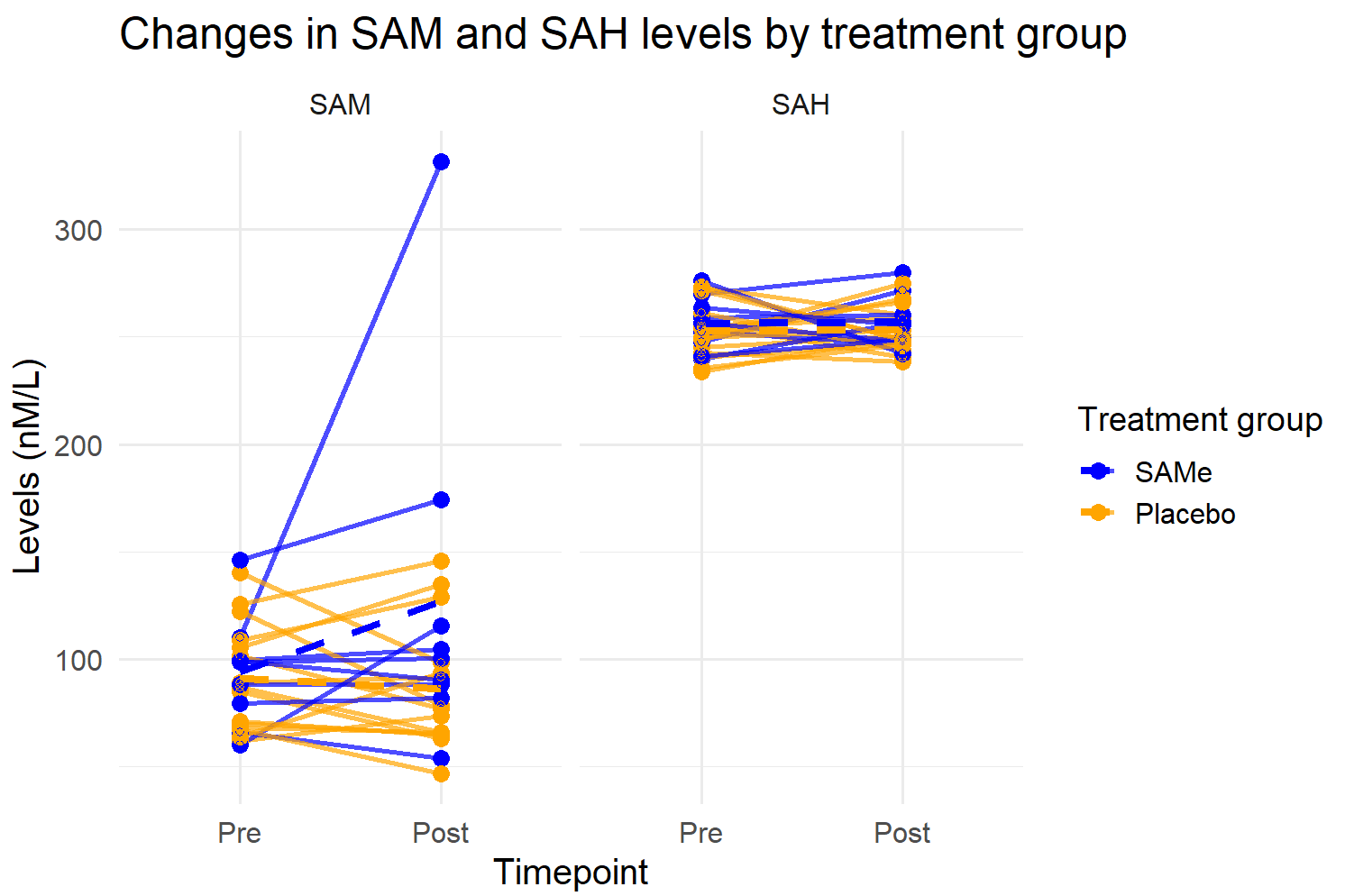


**Figure S1:** Changes in SAM and SAH plasma levels from pre- to post-treatment. The SAM plasma levels are depicted on the left, the SAH plasma levels on the right. Blue dots and lines correspond to the SAMe treatment group, the orange dots and lines to the placebo group. SAM= S-Adenosylmethionine; SAH= S-Adenosylhomocysteine; Pre= pre-treatment; Post= post-treatment.

**Table S1:** Epigenome wide association study results from the model *methylation post-treatment ~ methylation pre-treatment + randomization group + gender + cell count changes*, displaying the top 100 probes. CpG= 5'-C-phosphate-G-3';Chr=chromosome; Est= estimate; SE=standard error; FDR= false discovery rate.

| CpG | Chr | Position | Closest gene | Est | SE | P-value | FDR |
| --- | --- | --- | --- | --- | --- | --- | --- |
| cg07028850 | chr10 | 118618244 | ENO4 | -0.36 | 0.05 | 1.71E-06 | 0.83 |
| cg25132931 | chr3 | 112934149 | BOC | -0.34 | 0.05 | 2.48E-06 | 0.83 |
| cg09790137 | chr14 | 21439440 |  | 0.63 | 0.09 | 4.14E-06 | 0.83 |
| cg16646626 | chr1 | 204558847 |  | -0.45 | 0.07 | 7.49E-06 | 0.83 |
| cg23759600 | chr1 | 19334771 |  | -0.30 | 0.05 | 9.94E-06 | 0.83 |
| cg01709975 | chr4 | 25847472 | SEL1L3 | -0.16 | 0.03 | 1.01E-05 | 0.83 |
| cg12903648 | chr1 | 157522570 | FCRL5 | -0.33 | 0.05 | 1.02E-05 | 0.83 |
| cg12165123 | chr3 | 121658010 | SLC15A2 | -0.54 | 0.09 | 1.07E-05 | 0.83 |
| cg23737355 | chr12 | 121962631 | KDM2B | -0.42 | 0.07 | 1.09E-05 | 0.83 |
| cg02887048 | chr2 | 11356318 | ROCK2 | -0.28 | 0.05 | 1.19E-05 | 0.83 |
| cg18588194 | chr11 | 30304009 |  | -0.57 | 0.09 | 1.21E-05 | 0.83 |
| cg14560699 | chr6 | 132987463 |  | -0.28 | 0.05 | 1.46E-05 | 0.83 |
| cg01383486 | chr16 | 85721561 | GINS2 | -0.45 | 0.07 | 1.49E-05 | 0.83 |
| cg09000138 | chr10 | 119106703 | PDZD8 | -0.37 | 0.06 | 1.60E-05 | 0.83 |
| cg14571192 | chr1 | 85156339 | SSX2IP | 0.55 | 0.09 | 1.61E-05 | 0.83 |
| cg23956190 | chr7 | 1514455 | INTS1 | -0.16 | 0.03 | 1.74E-05 | 0.83 |
| cg04480057 | chr2 | 3404792 | TRAPPC12 | -0.36 | 0.06 | 1.78E-05 | 0.83 |
| cg07061500 | chr6 | 159290520 |  | -0.65 | 0.11 | 1.96E-05 | 0.83 |
| cg11159353 | chr14 | 96710906 |  | -0.26 | 0.04 | 1.96E-05 | 0.83 |
| cg13051051 | chr2 | 181087556 |  | -0.18 | 0.03 | 2.04E-05 | 0.83 |
| cg26705986 | chr12 | 123464567 | OGFOD2; ARL6IP4 | -0.64 | 0.11 | 2.05E-05 | 0.83 |
| cg22000644 | chr11 | 4630294 | TRIM68 | -0.26 | 0.04 | 2.15E-05 | 0.83 |
| cg13527921 | chr9 | 100819928 | NANS | -0.78 | 0.13 | 2.32E-05 | 0.86 |
| cg15216844 | chrX | 122906025 |  | -0.23 | 0.04 | 2.44E-05 | 0.87 |
| cg16494916 | chr19 | 7580945 | ZNF358 | -0.38 | 0.07 | 2.76E-05 | 0.90 |
| cg00018010 | chr17 | 20105626 | CYTSB | -0.25 | 0.04 | 2.77E-05 | 0.90 |
| cg02135728 | chr3 | 97486613 | ARL6 | -0.34 | 0.06 | 2.83E-05 | 0.90 |
| cg23408615 | chr19 | 1552315 |  | -0.30 | 0.05 | 2.99E-05 | 0.90 |
| cg06379435 | chr19 | 3344273 |  | -0.34 | 0.06 | 3.02E-05 | 0.90 |
| cg26381357 | chr12 | 55968117 | OR2AP1 | -0.45 | 0.08 | 3.20E-05 | 0.93 |
| cg19736660 | chr2 | 129050050 | HS6ST1 | -0.30 | 0.05 | 3.45E-05 | 0.95 |
| cg13549845 | chr4 | 93225960 | GRID2 | -0.36 | 0.06 | 3.57E-05 | 0.95 |
| cg01598285 | chr7 | 99154892 | ZNF655 | -0.30 | 0.05 | 3.58E-05 | 0.95 |
| cg13370900 | chr22 | 41777804 | TEF;TEF | 0.46 | 0.08 | 3.70E-05 | 0.95 |
| cg03444122 | chr4 | 189060646 | TRIML1 | -0.19 | 0.03 | 3.81E-05 | 0.95 |
| cg27364433 | chr10 | 97205920 | SORBS1 | -0.51 | 0.09 | 3.93E-05 | 0.95 |
| cg11723782 | chr3 | 159726973 | IL12A-AS1 | 0.28 | 0.05 | 4.31E-05 | 0.95 |
| cg20299414 | chr3 | 29447628 | RBMS3 | 0.30 | 0.05 | 4.32E-05 | 0.95 |
| cg06629493 | chr21 | 45788887 | TRPM2 | -0.26 | 0.05 | 4.60E-05 | 0.95 |
| cg15642209 | chr17 | 66290505 | ARSG | -0.20 | 0.04 | 4.91E-05 | 0.95 |
| cg17601941 | chr19 | 5889756 |  | -0.33 | 0.06 | 5.09E-05 | 0.95 |
| cg20702205 | chr7 | 5731340 | RNF216 | -0.26 | 0.05 | 5.13E-05 | 0.95 |
| cg02940523 | chr5 | 176264567 | UNC5A | -0.55 | 0.10 | 5.19E-05 | 0.95 |
| cg00643864 | chr10 | 115855613 |  | -0.66 | 0.12 | 5.33E-05 | 0.95 |
| cg13942156 | chr16 | 85176366 | LOC400548 | -0.29 | 0.05 | 5.43E-05 | 0.95 |
| cg19681528 | chr10 | 69644405 | SIRT1 | -0.29 | 0.05 | 5.49E-05 | 0.95 |
| cg06583259 | chr11 | 118758992 | CXCR5 | -0.44 | 0.08 | 5.52E-05 | 0.95 |
| cg02332206 | chr3 | 47933600 | MAP4 | -0.33 | 0.06 | 5.69E-05 | 0.95 |
| cg22726140 | chr14 | 22600032 |  | -0.28 | 0.05 | 6.04E-05 | 0.95 |
| cg09338678 | chr2 | 97576207 | FAM178B | -0.27 | 0.05 | 6.34E-05 | 0.95 |
| cg24944231 | chr5 | 34933675 | DNAJC21 | -0.24 | 0.05 | 6.45E-05 | 0.95 |
| cg09693631 | chr10 | 97453851 | TCTN3 | 0.43 | 0.08 | 6.51E-05 | 0.95 |
| cg23623851 | chr3 | 148992037 |  | -0.27 | 0.05 | 6.68E-05 | 0.95 |
| cg12661217 | chr2 | 81426434 |  | -0.31 | 0.06 | 6.70E-05 | 0.95 |
| cg17923478 | chr6 | 112853328 |  | -0.38 | 0.07 | 6.82E-05 | 0.95 |
| cg17705814 | chr20 | 36150135 | BLCAP; NNAT | -0.63 | 0.12 | 6.94E-05 | 0.95 |
| cg25790723 | chr12 | 94010838 |  | -0.34 | 0.06 | 7.04E-05 | 0.95 |
| cg21067858 | chr8 | 83074297 |  | -0.31 | 0.06 | 7.07E-05 | 0.95 |
| cg23500444 | chr13 | 99989271 | UBAC2 | -0.28 | 0.05 | 7.14E-05 | 0.95 |
| cg02286622 | chr18 | 8141323 | PTPRM | -0.24 | 0.04 | 7.14E-05 | 0.95 |
| cg06061466 | chr9 | 131815469 | FAM73B | -0.32 | 0.06 | 7.22E-05 | 0.95 |
| cg25444236 | chr13 | 96329629 | DNAJC3 | -0.42 | 0.08 | 7.33E-05 | 0.95 |
| cg14477708 | chr13 | 98702639 |  | -0.29 | 0.05 | 7.60E-05 | 0.95 |
| cg20650823 | chr14 | 102946182 | TECPR2 | -0.19 | 0.04 | 7.69E-05 | 0.95 |
| cg15851320 | chr7 | 129846204 | TMEM209; SSMEM1 | -0.29 | 0.05 | 7.73E-05 | 0.95 |
| cg07031798 | chr6 | 35766061 | CLPS | -0.36 | 0.07 | 8.05E-05 | 0.96 |
| cg12450316 | chr10 | 48362170 | ZNF488 | -0.34 | 0.06 | 8.13E-05 | 0.96 |
| cg01537928 | chr3 | 101280610 | RG9MTD1 | 0.35 | 0.07 | 8.51E-05 | 0.97 |
| cg22275309 | chr11 | 72069985 | CLPB | -0.28 | 0.05 | 8.59E-05 | 0.97 |
| cg08717030 | chr5 | 76933163 | OTP | 1.03 | 0.20 | 8.59E-05 | 0.97 |
| cg03109908 | chr7 | 135195030 | CNOT4 | 0.49 | 0.10 | 8.74E-05 | 0.97 |
| cg11159417 | chr5 | 148822491 |  | -0.29 | 0.06 | 8.95E-05 | 0.97 |
| ch.7.3114411F | chr7 | 148859697 | ZNF398 | 0.58 | 0.11 | 9.11E-05 | 0.97 |
| cg11379605 | chr1 | 117109623 | CD58 | -0.33 | 0.06 | 9.22E-05 | 0.97 |
| cg05470134 | chr7 | 142529154 |  | -0.27 | 0.05 | 9.27E-05 | 0.97 |
| cg04493806 | chr19 | 19635145 | NDUFA13 | -0.37 | 0.07 | 9.36E-05 | 0.97 |
| cg22404242 | chr2 | 86668131 | KDM3A | 0.46 | 0.09 | 9.53E-05 | 0.97 |
| cg21988461 | chr4 | 88008667 | AFF1 | -0.24 | 0.05 | 9.69E-05 | 0.97 |
| cg06927807 | chr1 | 229056866 |  | -0.36 | 0.07 | 9.78E-05 | 0.97 |
| cg06262098 | chr7 | 5413872 | TNRC18 | -0.20 | 0.04 | 9.93E-05 | 0.97 |
| cg25040418 | chr3 | 77861475 |  | -0.31 | 0.06 | 9.98E-05 | 0.97 |
| cg15931870 | chr14 | 37225623 | SLC25A21 | -0.30 | 0.06 | 1.01E-04 | 0.97 |
| cg13921570 | chr2 | 74730534 | LOC151534; LBX2 | -0.48 | 0.09 | 1.05E-04 | 0.97 |
| cg16618493 | chr1 | 154978980 | ZBTB7B | -0.19 | 0.04 | 1.06E-04 | 0.97 |
| cg08378788 | chr17 | 1395864 | MYO1C | -0.34 | 0.07 | 1.08E-04 | 0.97 |
| cg26301215 | chr3 | 111371676 |  | -0.25 | 0.05 | 1.09E-04 | 0.97 |
| cg17770110 | chr8 | 74205233 | RPL7 | 0.52 | 0.10 | 1.12E-04 | 0.97 |
| cg00981107 | chr10 | 11187389 | CELF2 | 1.24 | 0.24 | 1.12E-04 | 0.97 |
| cg13468215 | chr8 | 20146900 | LZTS1-AS1 | -0.28 | 0.06 | 1.15E-04 | 0.97 |
| cg10908460 | chr2 | 176950736 |  | 0.57 | 0.11 | 1.18E-04 | 0.97 |
| cg17987306 | chr2 | 156922626 | LOC101929378; | -0.39 | 0.08 | 1.20E-04 | 0.97 |
| cg14777730 | chr1 | 168076667 | GPR161 | -0.20 | 0.04 | 1.20E-04 | 0.97 |
| cg15740121 | chr19 | 12847981 | ASNA1 | -0.55 | 0.11 | 1.21E-04 | 0.97 |
| cg09227466 | chr2 | 233338248 |  | -0.29 | 0.06 | 1.23E-04 | 0.97 |
| cg11881202 | chr14 | 73712967 | PAPLN | -0.51 | 0.10 | 1.29E-04 | 0.97 |
| cg07387322 | chr6 | 88051717 | SMIM8 | -0.30 | 0.06 | 1.29E-04 | 0.97 |
| cg15712417 | chr14 | 52110206 | FRMD6 | -0.26 | 0.05 | 1.30E-04 | 0.97 |
| cg14609106 | chr13 | 22177940 | EFHA1 | -0.41 | 0.08 | 1.31E-04 | 0.97 |
| cg01900772 | chr2 | 170820946 | UBR3 | -0.25 | 0.05 | 1.36E-04 | 0.97 |
| cg26842121 | chr13 | 95358205 |  | 0.34 | 0.07 | 1.39E-04 | 0.97 |

**Table S2:** Sensitivity analysis results of epigenome wide association study, using the top quantile probes that captured the most variance, and adding number of diagnosis per participant as covariate to the model: *subset methylation post-treatment ~ subset methylation pre-treatment + randomization group + gender + delta cell counts + number of diagnoses.* CpG= 5'-C-phosphate-G-3';Chr=chromosome; Est= estimate; SE=standard error; FDR= false discovery rate.

| CpG | Chr | Position | Closest gene | Est | SE | P-value | FDR |
| --- | --- | --- | --- | --- | --- | --- | --- |
| cg17705814 | chr20 | 36150135 | BLCAP; NNAT | -0.85 | 0.10 | 3.71E-07 | 0.09 |
| cg22283921 | chr8 | 95961819 | TP53INP | 0.95 | 0.14 | 4.71E-06 | 0.36 |
| cg12302182 | chr15 | 95196461 |  | -0.43 | 0.07 | 1.93E-05 | 0.99 |
| cg16740022 | chr5 | 141806006 | LOC101926941 | -0.69 | 0.11 | 2.12E-05 | 0.99 |
| cg06379435 | chr19 | 3344273 |  | -0.39 | 0.07 | 3.26E-05 | 0.99 |
| cg26287345 | chr20 | 35169886 | MYL | 0.98 | 0.17 | 4.46E-05 | 0.99 |
| cg14739151 | chr2 | 154728234 | GALNT13 | -0.62 | 0.11 | 5.36E-05 | 0.99 |
| cg09790137 | chr14 | 21439440 |  | 0.56 | 0.10 | 5.76E-05 | 0.99 |
| cg14851485 | chr14 | 100126685 | HHIPL | -0.70 | 0.13 | 5.89E-05 | 0.99 |
| cg18277082 | chrX | 106599294 |  | -0.32 | 0.06 | 6.58E-05 | 0.99 |
| cg05624932 | chr8 | 75897310 | CRISPLD1 | -0.77 | 0.14 | 6.70E-05 | 0.99 |
| cg10417901 | chr1 | 110699480 | SLC6A17 | -0.54 | 0.10 | 7.01E-05 | 0.99 |
| cg15535221 | chr16 | 56554068 | BBS2 | -0.85 | 0.16 | 8.37E-05 | 0.99 |
| cg02940523 | chr5 | 176264567 | UNC5A | -0.65 | 0.12 | 8.46E-05 | 0.99 |
| cg08576928 | chr11 | 74859223 |  | -0.40 | 0.08 | 9.34E-05 | 0.99 |
| cg04757499 | chr10 | 86016597 | RGR | -0.36 | 0.07 | 1.03E-04 | 0.99 |
| cg27592523 | chr17 | 8024253 | HES7 | -0.85 | 0.16 | 1.04E-04 | 0.99 |
| cg16499645 | chr8 | 145654854 | VPS28; NFKBIL2 | -0.65 | 0.13 | 1.68E-04 | 0.99 |
| cg02100819 | chr19 | 29284511 |  | -0.40 | 0.08 | 1.71E-04 | 0.99 |
| cg25692524 | chr13 | 52769662 | THSD1P | -1.15 | 0.23 | 1.77E-04 | 0.99 |
| cg03851427 | chrX | 68399473 | LINC00269 | -0.43 | 0.09 | 1.89E-04 | 0.99 |
| cg07061500 | chr6 | 159290520 |  | -0.65 | 0.13 | 1.96E-04 | 0.99 |
| cg26705986 | chr12 | 123464567 | OGFOD2; ARL6IP4 | -0.62 | 0.13 | 2.14E-04 | 0.99 |
| cg05642143 | chr6 | 42928409 | GNMT | -0.95 | 0.20 | 2.15E-04 | 0.99 |
| cg13879776 | chr3 | 170136263 | CLDN11 | -0.92 | 0.19 | 2.17E-04 | 0.99 |
| cg16904960 | chr2 | 182549928 |  | -0.73 | 0.15 | 2.17E-04 | 0.99 |
| cg23744182 | chr6 | 28494196 | GPX5 | -0.40 | 0.08 | 2.20E-04 | 0.99 |
| cg26939341 | chr16 | 17626225 |  | -0.52 | 0.11 | 2.22E-04 | 0.99 |
| cg17866778 | chr6 | 26233442 |  | -0.34 | 0.07 | 2.46E-04 | 0.99 |
| cg24455236 | chr17 | 40824361 | PLEKHH3 | -0.82 | 0.17 | 2.51E-04 | 0.99 |
| cg20823481 | chr4 | 185736247 | ACSL1 | -0.42 | 0.09 | 2.53E-04 | 0.99 |
| cg17813054 | chrX | 92924956 |  | -0.40 | 0.08 | 2.69E-04 | 0.99 |
| cg17855437 | chr12 | 102009649 | MYBPC1 | -0.48 | 0.10 | 2.74E-04 | 0.99 |
| cg06914837 | chr4 | 186318163 | ANKRD37 | 0.66 | 0.14 | 2.79E-04 | 0.99 |
| cg19538644 | chr14 | 96644435 |  | -0.73 | 0.16 | 2.90E-04 | 0.99 |
| cg01383486 | chr16 | 85721561 | GINS2 | -0.37 | 0.08 | 3.10E-04 | 0.99 |
| cg22307470 | chrX | 149107867 | CXorf40B; LINC00894 | -0.57 | 0.12 | 3.23E-04 | 0.99 |
| cg16323491 | chr10 | 74032730 | DDIT4 | 0.65 | 0.14 | 3.31E-04 | 0.99 |
| cg05795649 | chrX | 9002177 | FAM9B | -0.82 | 0.18 | 3.53E-04 | 0.99 |
| cg13527921 | chr9 | 100819928 | NANS | -0.75 | 0.16 | 3.61E-04 | 0.99 |
| cg02538330 | chrX | 47004146 | NDUFB11; RBM10 | 0.54 | 0.12 | 3.65E-04 | 0.99 |
| cg09930074 | chr14 | 23208710 |  | -0.60 | 0.13 | 3.65E-04 | 0.99 |
| cg14028656 | chr20 | 42702150 |  | -0.41 | 0.09 | 3.66E-04 | 0.99 |
| cg15912732 | chr14 | 105255285 | AKT1 | 0.52 | 0.11 | 3.79E-04 | 0.99 |
| cg27364433 | chr10 | 97205920 | SORBS1 | -0.47 | 0.10 | 3.80E-04 | 0.99 |
| cg11530693 | chr1 | 120165357 | ZNF697 | -0.46 | 0.10 | 3.80E-04 | 0.99 |
| cg02611169 | chr17 | 80334617 |  | -0.23 | 0.05 | 3.82E-04 | 0.99 |
| cg03233972 | chr16 | 87167395 |  | 0.53 | 0.12 | 3.93E-04 | 0.99 |
| cg08717030 | chr5 | 76933163 | OTP | 1.02 | 0.23 | 4.04E-04 | 0.99 |
| cg01431340 | chr6 | 110680085 | C6orf186 | -0.48 | 0.11 | 4.07E-04 | 0.99 |
| cg09208213 | chr5 | 131991700 | TH2LCRR | -0.59 | 0.13 | 4.20E-04 | 0.99 |
| cg05212510 | chr18 | 77623544 | KCNG2 | 1.25 | 0.28 | 4.31E-04 | 0.99 |
| cg16620673 | chr13 | 19979258 |  | -0.55 | 0.12 | 4.36E-04 | 0.99 |
| cg14998184 | chr20 | 61464108 | COL9A3 | 0.87 | 0.20 | 4.55E-04 | 0.99 |
| cg15571561 | chr3 | 35706161 | ARPP-21 | -0.74 | 0.17 | 4.55E-04 | 0.99 |
| cg27555056 | chr2 | 207629829 | MDH1B; FASTKD2 | -0.71 | 0.16 | 4.78E-04 | 0.99 |
| cg21462844 | chr8 | 67628165 | SGK3 | -0.59 | 0.13 | 4.81E-04 | 0.99 |
| cg14998497 | chr12 | 124229435 | ATP6V0A2 | 0.49 | 0.11 | 4.88E-04 | 0.99 |
| cg00643864 | chr10 | 115855613 |  | -0.64 | 0.14 | 4.92E-04 | 0.99 |
| cg23509665 | chr7 | 158293697 | PTPRN2 | -0.71 | 0.16 | 5.00E-04 | 0.99 |
| cg21821214 | chr2 | 176969223 |  | -0.87 | 0.20 | 5.17E-04 | 0.99 |
| cg12973365 | chr21 | 38353318 | HLCS | -0.83 | 0.19 | 5.23E-04 | 0.99 |
| cg02396192 | chr7 | 154858549 |  | -0.48 | 0.11 | 5.26E-04 | 0.99 |
| cg22221557 | chr2 | 33308864 | LTBP1 | -0.68 | 0.16 | 5.28E-04 | 0.99 |
| cg14129931 | chr18 | 74513950 |  | -0.70 | 0.16 | 5.41E-04 | 0.99 |
| cg09095403 | chr22 | 26859981 | HPS4 | -0.25 | 0.06 | 5.42E-04 | 0.99 |
| cg13202578 | chr1 | 26081363 | MAN1C1 | -0.33 | 0.08 | 5.54E-04 | 0.99 |
| cg19611807 | chr14 | 58705466 |  | -0.83 | 0.19 | 5.71E-04 | 0.99 |
| cg24671636 | chr11 | 7244094 |  | -0.53 | 0.12 | 5.71E-04 | 0.99 |
| cg03350138 | chr6 | 28446794 |  | -0.42 | 0.10 | 5.84E-04 | 0.99 |
| cg13921570 | chr2 | 74730534 | LOC151534; LBX2 | -0.49 | 0.11 | 5.93E-04 | 0.99 |
| cg21757178 | chr6 | 24745355 |  | -0.36 | 0.08 | 5.97E-04 | 0.99 |
| cg12219752 | chr9 | 72000277 | FAM189A2 | -0.84 | 0.19 | 5.98E-04 | 0.99 |
| cg13468215 | chr8 | 20146900 | LZTS1-AS1 | -0.29 | 0.07 | 6.06E-04 | 0.99 |
| cg09496273 | chr2 | 241303563 |  | 0.41 | 0.09 | 6.06E-04 | 0.99 |
| cg26120960 | chr3 | 47918946 | MAP4 | -0.52 | 0.12 | 6.14E-04 | 0.99 |
| cg20451272 | chr9 | 131155135 | MIR219A2; MIR1268A | 0.66 | 0.15 | 6.16E-04 | 0.99 |
| cg00035847 | chr1 | 29557016 | MECR | -0.45 | 0.10 | 6.19E-04 | 0.99 |
| cg16001418 | chr19 | 36393325 | HCST | -1.26 | 0.29 | 6.32E-04 | 0.99 |
| cg19901956 | chr11 | 77921274 | USP35 | -0.68 | 0.16 | 6.32E-04 | 0.99 |
| cg00118309 | chrX | 76141257 | MIR384; MIR325HG | -0.45 | 0.10 | 6.43E-04 | 0.99 |
| cg05382653 | chr8 | 93891273 |  | 0.78 | 0.18 | 6.47E-04 | 0.99 |
| cg27039118 | chr8 | 116575902 | TRPS1 | -0.47 | 0.11 | 6.53E-04 | 0.99 |
| cg05945782 | chr17 | 1954986 | MIR212 | -0.71 | 0.17 | 6.58E-04 | 0.99 |
| cg01414824 | chr5 | 52160870 | ITGA1 | -0.72 | 0.17 | 6.58E-04 | 0.99 |
| cg08112724 | chr14 | 88788364 | KCNK10 | -0.58 | 0.13 | 6.62E-04 | 0.99 |
| cg13912090 | chr5 | 95767863 | PCSK1 | -0.41 | 0.10 | 6.65E-04 | 0.99 |
| cg21031875 | chr10 | 101691159 | DNMBP; DNMBP-AS1 | -0.41 | 0.10 | 6.75E-04 | 0.99 |
| cg02940147 | chr11 | 124747263 | ROBO3 | -0.54 | 0.13 | 6.88E-04 | 0.99 |
| cg02644111 | chr2 | 160313254 | BAZ2B; | -0.51 | 0.12 | 7.01E-04 | 0.99 |
| cg07853937 | chr10 | 99168546 |  | -0.42 | 0.10 | 7.04E-04 | 0.99 |
| cg21772629 | chrX | 99668480 |  | -0.40 | 0.10 | 7.07E-04 | 0.99 |
| cg16654102 | chr11 | 73796780 | C2CD3 | -0.44 | 0.10 | 7.12E-04 | 0.99 |
| cg10734892 | chr8 | 27468923 | CLU; MIR6843 | -0.43 | 0.10 | 7.19E-04 | 0.99 |
| cg17281355 | chrX | 153738942 | FAM3A | -0.45 | 0.11 | 7.31E-04 | 0.99 |
| cg00981107 | chr10 | 11187389 | CELF2 | 1.24 | 0.29 | 7.56E-04 | 0.99 |
| cg24135868 | chr5 | 142217046 | ARHGAP26 | -0.25 | 0.06 | 7.65E-04 | 0.99 |
| cg21330407 | chrX | 100548103 | TAF7L | -0.52 | 0.12 | 7.76E-04 | 0.99 |
| cg06708107 | chr7 | 136641675 | CHRM2; LOC349160 | -0.38 | 0.09 | 7.77E-04 | 0.99 |
| cg16839933 | chrX | 47696695 | ZNF81 | -0.39 | 0.09 | 7.87E-04 | 0.99 |

**Table S3:** Differentially Methylated Regions (DMRs) derived from epigenome wide association study testing the effect of SAMe intervention on methylation levels using the model *methylation post-treatment ~ methylation pre-treatment + randomization group + gender + cell count changes*. Chr=chromosome; CpG= 5'-C-phosphate-G-3'; n=number; SE=standard error; Adj=adjusted.

|  | Region (chr:start-end) | CpGs (n) | Effect size (B) | SE | P-value | Adj. p-value |
| --- | --- | --- | --- | --- | --- | --- |
| 1 | chr6:3849391-3849818 | 11 | -0.42 | 0.04 | 1.14E-25 | 8.67E-20 |
| 2 | chr11:67372443-67373114 | 6 | -0.45 | 0.06 | 3.93E-16 | 3E-10 |
| 3 | chr14:96710013-96710906 | 5 | -1.09 | 0.14 | 1.18E-15 | 9E-10 |
| 4 | chr6:43478215-43478692 | 8 | -0.42 | 0.05 | 6.14E-15 | 4.68E-09 |
| 5 | chr20:3065343-3065698 | 8 | -0.39 | 0.05 | 1.11E-14 | 8.46E-09 |
| 6 | chr17:1395371-1396298 | 8 | -0.42 | 0.06 | 1.85E-14 | 1.41E-08 |
| 7 | chr6:123316988-123317569 | 11 | -0.66 | 0.09 | 2.43E-14 | 1.85E-08 |
| 8 | chr19:7580857-7580945 | 3 | -0.52 | 0.07 | 8.69E-14 | 6.63E-08 |
| 9 | chr2:154728210-154728234 | 2 | -1.16 | 0.16 | 2.18E-13 | 1.66E-07 |
| 10 | chr5:140186484-140186895 | 7 | -0.53 | 0.07 | 4.88E-13 | 3.72E-07 |
| 11 | chr14:39644224-39644624 | 7 | -0.53 | 0.07 | 1.08E-12 | 8.25E-07 |
| 12 | chr6:29521145-29521501 | 10 | -0.75 | 0.11 | 4.00E-12 | 3.05E-06 |
| 13 | chr10:22331462-22331872 | 2 | -0.46 | 0.07 | 4.64E-12 | 3.54E-06 |
| 14 | chr2:27665128-27665711 | 8 | -0.70 | 0.10 | 7.90E-12 | 6.02E-06 |
| 15 | chr17:8213604-8214081 | 4 | -1.23 | 0.18 | 7.90E-12 | 6.02E-06 |
| 16 | chr2:210444075-210444270 | 6 | -1.18 | 0.17 | 8.94E-12 | 6.82E-06 |
| 17 | chr2:20386466-20386757 | 4 | -0.50 | 0.07 | 9.21E-12 | 7.02E-06 |
| 18 | chr14:101528332-101528354 | 2 | -0.51 | 0.08 | 1.24E-11 | 9.47E-06 |
| 19 | chr15:45421612-45422083 | 10 | -0.50 | 0.07 | 2.23E-11 | 1.7E-05 |
| 20 | chr6:42928409-42928920 | 11 | -0.63 | 0.09 | 2.40E-11 | 1.83E-05 |
| 21 | chr8:57233269-57233314 | 3 | -0.70 | 0.11 | 4.56E-11 | 3.47E-05 |
| 22 | chr5:147052374-147052498 | 2 | -1.58 | 0.24 | 6.79E-11 | 5.18E-05 |
| 23 | chr10:3138418-3138534 | 3 | -1.19 | 0.18 | 9.31E-11 | 7.1E-05 |
| 24 | chr8:141580048-141580128 | 2 | -0.56 | 0.09 | 1.05E-10 | 7.99E-05 |
| 25 | chr15:75018200-75018700 | 11 | -0.41 | 0.06 | 1.19E-10 | 9.05E-05 |
| 26 | chr10:118083742-118084320 | 6 | -0.54 | 0.08 | 1.27E-10 | 9.67E-05 |
| 27 | chr6:170863290-170863483 | 6 | -0.51 | 0.08 | 1.33E-10 | <0.00 |
| 28 | chr17:7283774-7284049 | 7 | -0.50 | 0.08 | 1.77E-10 | <0.00 |
| 29 | chr19:41256731-41257117 | 5 | 0.83 | 0.13 | 2.23E-10 | <0.00 |
| 30 | chr16:56554068-56554074 | 2 | -1.31 | 0.21 | 3.00E-10 | <0.00 |
| 31 | chr6:28446794-28447107 | 4 | -1.14 | 0.18 | 3.89E-10 | <0.00 |
| 32 | chr7:135242290-135242568 | 6 | 0.59 | 0.09 | 4.59E-10 | <0.00 |
| 33 | chr13:99135543-99135770 | 6 | -0.71 | 0.11 | 4.87E-10 | <0.00 |
| 34 | chr16:25114920-25115053 | 2 | -1.17 | 0.19 | 7.86E-10 | <0.00 |
| 35 | chr2:170820946-170821095 | 2 | -0.61 | 0.10 | 8.97E-10 | <0.00 |
| 36 | chr16:57406074-57406395 | 5 | -0.93 | 0.15 | 1.01E-09 | <0.00 |
| 37 | chr3:47933600-47933936 | 2 | -0.92 | 0.15 | 1.17E-09 | <0.00 |
| 38 | chr8:131386946-131387399 | 2 | -0.59 | 0.10 | 1.51E-09 | <0.00 |
| 39 | chr1:1356728-1356921 | 6 | -0.51 | 0.08 | 1.58E-09 | <0.00 |
| 40 | chr1:85156339-85156424 | 3 | 0.79 | 0.13 | 2.55E-09 | <0.00 |
| 41 | chr20:47895017-47895299 | 6 | 0.44 | 0.07 | 2.70E-09 | <0.00 |
| 42 | chr16:3068085-3068529 | 8 | -0.55 | 0.09 | 2.76E-09 | <0.00 |
| 43 | chr5:131991700-131991765 | 2 | -0.68 | 0.11 | 3.08E-09 | <0.00 |
| 44 | chr5:115786119-115786511 | 3 | -0.70 | 0.12 | 3.32E-09 | <0.00 |
| 45 | chr6:35766051-35766061 | 2 | -0.76 | 0.13 | 5.40E-09 | <0.00 |
| 46 | chr20:35169846-35169886 | 3 | 1.14 | 0.20 | 6.91E-09 | 0.01 |
| 47 | chr2:144895454-144895805 | 2 | -1.04 | 0.18 | 8.69E-09 | 0.01 |
| 48 | chr12:12938082-12938425 | 6 | -0.42 | 0.07 | 1.14E-08 | 0.01 |
| 49 | chr14:78869751-78870232 | 6 | -0.53 | 0.10 | 2.07E-08 | 0.02 |
| 50 | chr19:5799340-5799467 | 3 | -0.72 | 0.13 | 2.21E-08 | 0.02 |
| 51 | chr22:43505954-43506327 | 8 | -0.56 | 0.10 | 2.38E-08 | 0.02 |
| 52 | chr5:38445614-38445996 | 5 | -0.49 | 0.09 | 2.43E-08 | 0.02 |
| 53 | chr1:165414604-165414806 | 4 | -1.25 | 0.22 | 2.50E-08 | 0.02 |
| 54 | chr1:25257587-25257599 | 2 | -0.91 | 0.16 | 2.79E-08 | 0.02 |
| 55 | chr20:18491484-18491545 | 3 | -0.55 | 0.10 | 3.01E-08 | 0.02 |
| 56 | chr19:2255419-2255744 | 7 | -0.75 | 0.13 | 3.07E-08 | 0.02 |
| 57 | chr12:111021254-111021265 | 2 | 0.98 | 0.18 | 3.07E-08 | 0.02 |
| 58 | chr9:131815078-131815469 | 2 | -0.42 | 0.08 | 3.17E-08 | 0.02 |
| 59 | chrX:68399327-68399473 | 3 | -0.74 | 0.13 | 3.70E-08 | 0.03 |
| 60 | chr17:79872318-79872369 | 2 | -0.71 | 0.13 | 4.24E-08 | 0.03 |
| 61 | chr4:74847758-74847829 | 5 | -0.69 | 0.13 | 4.64E-08 | 0.04 |
| 62 | chr5:6660096-6660406 | 2 | -0.87 | 0.16 | 4.77E-08 | 0.04 |
| 63 | chr6:33141152-33141824 | 5 | -0.54 | 0.10 | 5.12E-08 | 0.04 |
| 64 | chr17:872770-873452 | 4 | -0.86 | 0.16 | 5.30E-08 | 0.04 |
| 65 | chr15:74495276-74495603 | 5 | -0.66 | 0.12 | 5.77E-08 | 0.04 |
| 66 | chr16:1048006-1048327 | 4 | -0.85 | 0.16 | 6.44E-08 | 0.05 |

**Table S4:** Results from gene ontology (GO) term enrichment analysis, with annotated differentially methylated probes (DMPs) as target genes and the genes at Illumina EPIC array as reference. The GO term analysis was performed with CpGs ranked from the lowest to highest FDR corrected p-value. Displayed are the GO terms with FDR q-value <0.05 and enrichment value >3.

| **GO Term** | **Description** | **P-value** | **FDR q-value** | **Enrichment** | **Genes** |
| --- | --- | --- | --- | --- | --- |
| GO:0007156 | homophilic cell adhesion via plasma membrane adhesion molecules | 5.26E-30 | 8.16E-26 | 10.31 | FAT1, CDH4, PCDHGA10, PCDHGA9, PTPRM, PCDHGA7, PCDHGB4, PCDHGA6, DCHS1, PCDHGA5, PCDHGA3, PCDHGA2, PCDHGA8, SDK1, CDH22, PCDHAC1, PCDHA13, PCDHA12, PCDHA11, PCDHA10, PCDHA8, PCDHA7, PCDHA6, PCDHA5, PCDHA4, PCDHA3, ROBO3, PCDHA2, PCDHA1, PCDHGA12, PCDHA9, PCDHGC5, PCDHGC4, PCDHGB7, PCDHGB6, PCDHGC3, PCDHGB5, PCDHGB3, PCDHGB2, PCDHGB1, PCDHGA11 |
| GO:0098742 | cell-cell adhesion via plasma-membrane adhesion molecules | 9.37E-25 | 7.26E-21 | 6.53 | PCDHGA10, FAT1, PCDHGA9, PTPRM, PCDHGA7, PCDHGA6, PCDHGB4, PCDHGA5, DCHS1, PCDHGA4, PCDHGA3, PTPRS, PCDHGA2, PCDHGA1, CDH22, PCDHAC1, PCDHA13, PCDHA12, PCDHA11, PCDHA10, PCDHA8, PCDHA7, PCDHA6, PCDHA5, PCDHA4, PCDHA3, PCDHA2, PCDHA1, DSCAML1, PCDHGC3, CDH2, CDH4, CLDN12, PCDHGA8, SDK1, GRID2, TENM4, ROBO3, PCDHGA12, PCDHA9, CLDN11, PCDHGC5, PCDHGC4, PCDHGB7, PCDHGB6, PCDHGB5, PCDHGB3, PTPRF, PCDHGB2, PCDHGB1, PCDHGA11 |
| GO:0098609 | cell-cell adhesion | 2.49E-16 | 1.29E-12 | 3.58 | PCDHGA10, FAT1, PCDHGA9, PCDHGA7, PTPRM, PCDHGA6, PCDHGB4, PCDHGA5, DCHS1, PCDHGA4, PCDHGA3, PCDHGA2, PTPRS, PCDHGA1, CDH22, PCDHAC2, CD44, PCDHAC1, PCDHA13, PCDHA12, MYL9, PCDHA11, CD58, PCDHA10, PCDHA8, PCDHA7, PCDHA6, PCDHA5, PCDHA4, PCDHA3, PCDHA2, PCDHA1, NRXN3, ZFP36L1, DSCAML1, PCDHGC3, EGFR, CDH2, CDH4, CLDN12, TJP1, PCDHGA8, SDK1, GRID2, TENM4, ROBO3, PCDHGA12, VCL, ITGA7, PKHD1, PCDHA9, CLDN11, ITGB3, PCDHGC5, PCDHGC4, PCDHGB7, PCDHGB6, PCDHGB5, PCDHGB3, PCDHGB2, LPP, PTPRF, PCDHGB1, PCDHGA11 |
| GO:0007155 | cell adhesion | 1.11E-13 | 4.28E-10 | 3.03 | PCDHGA10, FAT1, PCDHGA9, PTPRM, PCDHGA7, FBLN5, DDR1, PCDHGB4, PCDHGA6, DCHS1, PCDHGA5, PCDHGA4, PCDHGA3, PCDHGA2, PCDHGA1, ENTPD1, LY9, CD44, PCDHAC1, PCDHA13, PCDHA12, CD58, PCDHA11, COL4A3, PCDHA10, PCDHA8, PCDHA7, PCDHA6, PCDHA5, PCDHA4, PCDHA3, PCDHA2, PCDHA1, LAMB3, ZFP36L1, COL12A1, COL16A1, PCDHGC3, SORBS1, CDH2, CDH4, CLDN12, ADAMTS12, LRRN2, PCDHGA8, SDK1, SSX2IP, BOC, GRID2, PTK7, ROBO3, ITGA7, PCDHGA12, VCL, ITGAE, ANGPT1, PKHD1, PCDHA9, CLDN11, PCDHGC5, PCDHGC4, PCDHGB7, PCDHGB6, PCDHGB5, PCDHGB3, PCDHGB2, LPP, PCDHGB1, PCDHGA11 |
| GO:0022610 | biological adhesion | 1.56E-13 | 4.83E-10 | 3.01 | PCDHGA10, FAT1, PCDHGA9, PTPRM, PCDHGA7, FBLN5, DDR1, PCDHGB4, PCDHGA6, DCHS1, PCDHGA5, PCDHGA4, PCDHGA3, PCDHGA2, PCDHGA1, ENTPD1, LY9, CD44, PCDHAC1, PCDHA13, PCDHA12, CD58, PCDHA11, COL4A3, PCDHA10, PCDHA8, PCDHA7, PCDHA6, PCDHA5, PCDHA4, PCDHA3, PCDHA2, PCDHA1, LAMB3, ZFP36L1, COL12A1, COL16A1, PCDHGC3, SORBS1, CDH2, CDH4, CLDN12, ADAMTS12, LRRN2, PCDHGA8, SDK1, SSX2IP, BOC, GRID2, PTK7, ROBO3, ITGA7, PCDHGA12, VCL, ITGAE, ANGPT1, PKHD1, PCDHA9, CLDN11, PCDHGC5, PCDHGC4, PCDHGB7, PCDHGB6, PCDHGB5, PCDHGB3, PCDHGB2, LPP, PCDHGB1, PCDHGA11 |
| GO:1900078 | positive regulation of cellular response to insulin stimulus | 6.24E-6 | 2.31E-3 | 9.95 | ZBTB7B, PRKCZ, SERPINA12, MYO1C, PAK1, SIRT1, SORBS1, GNAI2 |
| GO:0032012 | regulation of ARF protein signal transduction | 8.09E-6 | 2.85E-3 | 5.23 | CYTH2, CYTH1, KIAA1244, MAP4K4, FBXO8, PSD3, IQSEC1, PSD4, GBF1, IQSEC2, ARFGEF2 |

**Table S5:** Results from gene ontology (GO) term enrichment analysis, with annotated differentially methylated regions (DMRs) as target genes and the genes at the Illumina EPIC array as reference. Displayed are all GO terms for biological process, no GO terms were found for cellular components and function.

| **GO Term** | **Description** | **P-value** | **FDR q-value** | **Enrichment** | **Genes** |
| --- | --- | --- | --- | --- | --- |
| GO:0016101 | diterpenoid metabolic process | 3.02E-5 | 4.69E-1 | 13.87 | SRD5A1, STRA6, CLPS, CYP1A1 |
| GO:0006721 | terpenoid metabolic process | 4.52E-5 | 3.51E-1 | 12.75 | SRD5A1, STRA6, SDR16C5, CLPS, CYP1A1 |
| GO:0006720 | isoprenoid metabolic process | 8.06E-5 | 4.17E-1 | 11.3 | SRD5A1, STRA6, SDR16C5, CLPS, CYP1A1 |
| GO:0042310 | vasoconstriction | 1.04E-4 | 4.02E-1 | 32.72 | BBS2, AVP, BDKRB2 |
| GO:0097756 | negative regulation of blood vessel diameter | 1.04E-4 | 3.22E-1 | 32.72 | BBS2, AVP, BDKRB2 |
| GO:0098609 | cell-cell adhesion | 1.87E-4 | 4.85E-1 | 4.99 | MYL9, NRXN3, PCDHA4, PCDHA3, CLDN6, PCDHA2, PCDHA1, CX3CL1 |
| GO:0042743 | hydrogen peroxide metabolic process | 2.54E-4 | 5.62E-1 | 24.33 | DUOXA1, DUOX1, CYP1A1 |
| GO:0050665 | hydrogen peroxide biosynthetic process | 3.49E-4 | 6.77E-1 | 70.29 | CYP1A1, DUOX1 |
| GO:0048731 | system development | 3.66E-4 | 6.32E-1 | 4.01 | SEMA6A, COL11A2, NXN, SRD5A1, PCDHA4, STRA6, PCDHA3, PCDHA2, PCDHA1 |
| GO:0001523 | retinoid metabolic process | 3.8E-4 | 5.89E-1 | 11.71 | STRA6, SDR16C5, CLPS, CYP1A1 |
| GO:0007155 | cell adhesion | 5.56E-4 | 7.84E-1 | 3.45 | MYL9, NRXN3, PCDHA4, PCDHA3, CLDN6, PCDHA2, PCDHA1, PNN, CX3CL1 |
| GO:0022610 | biological adhesion | 5.85E-4 | 7.56E-1 | 3.43 | MYL9, NRXN3, PCDHA4, PCDHA3, CLDN6, PCDHA2, PCDHA1, PNN, CX3CL1 |
| GO:0017000 | antibiotic biosynthetic process | 7.5E-4 | 8.95E-1 | 48.66 | DUOX1, CYP1A1 |
| GO:0030540 | female genitalia development | 8.73E-4 | 9.68E-1 | 45.18 | SRD5A1, STRA6 |


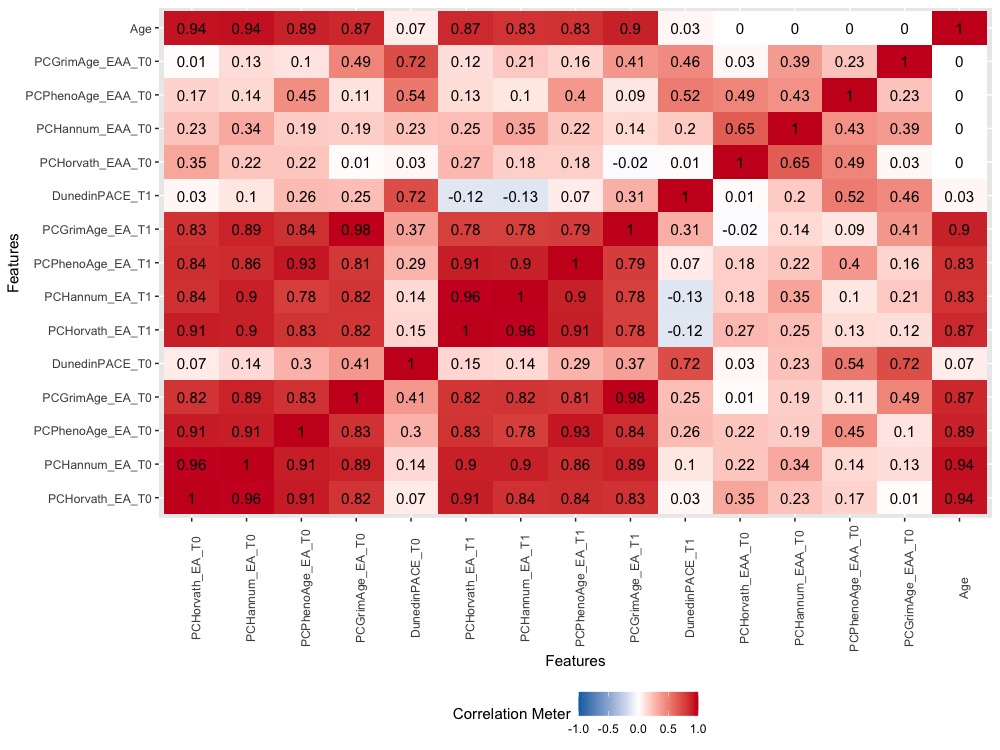
**Figure S2:** Correlation heatmap with principal component (PC) versions of four epigenetic clocks (PCHorvath, PCHannum, PCPhenoAge, PCGrimAge), the pace of aging clock DunedinPACE, and chronological age. EA= Epigenetic Age, EAA= Epigenetic Age Acceleration, T0=pre-treatment, T1=post-treatment.

**Table S6:** Effect estimates from linear regression analysis on whether SAMe influences epigenetic age changes over the treatment period, using the model *EA post-treatment ~ EA pre-treatment + randomization group + gender*.

|  | **PCHorvath** | | | **PCHannum** | | | **PCPhenoAge** | | | **PCGrimAge** | | | **DunedinPACE** | | |
| --- | --- | --- | --- | --- | --- | --- | --- | --- | --- | --- | --- | --- | --- | --- | --- |
| Adj. R^2^ | 0.81 | | | 0.80 | | | 0.84 | | | 0.96 | | | 0.47 | | |
| F | 37.17 | | | 35.25 | | | 47.91 | | | 229 | | | 8.733 | | |
| *p* | 5.48e-09** | | | 9.03e-09** | | | 4.71e-10** | | | 2.2e-16** | | | 0.00** | | |
|  | β | SE | *p* | β | SE | *p* | β | SE | *p* | β | SE | *p* | β | SE | *p* |
| Intercept | 4.40 | 4.72 | 0.36 | 0.25 | 5.98 | 0.97 | 0.24 | 4.40 | 0.96 | -0.46 | 2.94 | 0.88 | 0.22 | 0.16 | 0.19 |
| EA pre-treatment | 0.91 | 0.09 | 4.09e-10** | 0.99 | 0.10 | 8.87e-10** | 1.00 | 0.09 | 4.58e-11** | 1.01 | 0.04 | <2e-16** | 0.80 | 0.15 | 3.56e-05** |
| SAMe | 0.66 | 1.11 | 0.56 | 1.40 | 1.38 | 0.32 | 1.22 | 1.55 | 0.44 | -0.02 | 0.67 | 0.98 | -0.00 | 0.04 | 0.91 |
| Gender (F) | -0.80 | 1.10 | 0.48 | -1.01 | 1.34 | 0.46 | -0.59 | 1.50 | 0.70 | 0.90 | 0.68 | 0.20 | 0.03 | 0.04 | 0.51 |

Adj=adjusted; F=F-statistic; *p* = p-value; EA= Epigenetic Age; β= beta; SE=standard error; SAMe=S-adenosylmethionine; F=female. For all models, df= 23. *p<0.05, **p<0.01

**Table S7:** Effect estimates from linear regression analysis on association between epigenetic age acceleration and depressive symptoms pre-treatment, using the model *Depression symptoms baseline ~ EAA baseline + age + gender*.

|  | **PCHorvath** | | | **PCHannum** | | | **PCPhenoAge** | | | **PCGrimAge** | | | **DunedinPACE** | | |
| --- | --- | --- | --- | --- | --- | --- | --- | --- | --- | --- | --- | --- | --- | --- | --- |
| Adj. R^2^ | -0.07 | | | -0.07 | | | 0.05 | | | -0.02 | | | -0.03 | | |
| F | 0.46 | | | 0.41 | | | 1.43 | | | 0.83 | | | 0.74 | | |
| *p* | 0.71 | | | 0.75 | | | 0.26 | | | 0.49 | | | 0.54 | | |
|  | β | SE | *p* | β | SE | *p* | β | SE | *p* | β | SE | *p* | β | SE | *p* |
| Intercept | 15.65 | 4.55 | 0.00** | 15.70 | 4.56 | 0.00** | 16.69 | 4.34 | 0.00** | 14.97 | 4.50 | 0.00** | 8.85 | 8.36 | 0.30 |
| EAA pre-treatment | -0.16 | 0.41 | 0.70 | -0.01 | 0.39 | 0.99 | 0.39 | 0.23 | 0.10 | 0.25 | 0.23 | 0.29 | 6.81 | 7.04 | 0.34 |
| Age | 0.09 | 0.09 | 0.32 | 0.09 | 0.86 | 0.32 | 0.08 | 0.08 | 0.32 | 0.09 | 0.08 | 0.29 | 0.08 | 0.08 | 0.34 |
| Gender (F) | -0.61 | 1.83 | 0.74 | -0.67 | 1.83 | 0.72 | -1.90 | 1.87 | 0.32 | 0.23 | 1.97 | 0.91 | 0.51 | 1.80 | 0.778 |

Adj= adjusted; F=F-statistic; *p=* p-value; β = beta; SE= standard error; EAA= Epigenetic Age Acceleration; F=female. For all models, df= 23. *p<0.05, **p<0.01

**Table S8:** Effect estimates from linear regression analysis predicting change in depressive symptoms over the treatment period by baseline epigenetic age acceleration and change in epigenetic age, using the model *Changed depression symptoms ~ EAA pre-treatment + EA change + chronological age + gender*. For DunedinPACE, no change in epigenetic age was calculated as this clock already measures pace of aging.

|  | **PCHorvath** | | | **PCHannum** | | | **PCPhenoAge** | | | **PCGrimAge** | | | **DunedinPACE** | | |
| --- | --- | --- | --- | --- | --- | --- | --- | --- | --- | --- | --- | --- | --- | --- | --- |
| Adj. R2 | 0.11 | | | -0.02 | | | 0.00 | | | -0.10 | | | -0.08 | | |
| F | 2.12 | | | 0.80 | | | 1.02 | | | 0.18 | | | 0.09 | | |
| *p* | 0.12 | | | 0.51 | | | 0.40 | | | 0.91 | | | 0.91 | | |
|  | β | SE | *p* | β | SE | *p* | β | SE | *p* | β | SE | *p* | β | SE | *p* |
| Intercept | -6.93 | 2.33 | 0.01* | -7.43 | 2.50 | 0.01* | -6.49 | 2.59 | 0.02* | -6.77 | 2.75 | 0.02* | -3.08 | 13.78 | 0.83 |
| EAA pre-treatment | 1.74 | 0.69 | 0.02* | 0.93 | 0.69 | 0.19 | 0.55 | 0.42 | 0.21 | -0.19 | 0.46 | 0.69 | -3.95 | 13.04 | 0.76 |
| EA change | 0.26 | 0.56 | 0.64 | 0.27 | 0.49 | 0.59 | 0.51 | 0.44 | 0.25 | 0.44 | 1.13 | 0.70 | - | - | - |
| Gender (F) | 0.59 | 3.03 | 0.85 | 1.51 | 3.27 | 0.65 | -0.46 | 3.46 | 0.90 | -0.04 | 3.73 | 0.99 | 0.90 | 3.33 | 0.79 |

Adj= adjusted; F=F-statistic; *p=* p-value; β = beta; SE= standard error; EAA= Epigenetic Age Acceleration; EA= Epigenetic Age; F=female. For all models, df= 23. *p<0.05, **p<0.01
